# *CD38* is associated with communal behavior, partner perceptions, affect and relationship adjustment in romantic relationships

**DOI:** 10.1038/s41598-020-69520-y

**Published:** 2020-08-20

**Authors:** Gentiana Sadikaj, D. S. Moskowitz, David C. Zuroff, Jennifer A. Bartz

**Affiliations:** Department of Psychology, McGill University, 2001 McGill College Avenue, Montreal, QC H3A 1G1 Canada

**Keywords:** Neuroscience, Endocrinology

## Abstract

Given the significance of close relationships for human survival, it is thought that biological mechanisms evolved to support their initiation and maintenance. The neuropeptide oxytocin is one such candidate identified in non-human animal research. We investigated whether variation in *CD38*, a gene involved in oxytocin secretion and attachment behavior in rodents, predicts romantic relationship dynamics in daily life. Community couples participated in an event-contingent recording (ECR) study in which they reported their social behavior, perception of their partner’s behavior, and affect during their interactions with one another over a 20-day period; couples also completed various measures of relationship adjustment. Out of the 111 couples (N = 222 individuals) who provided either ECR and/or relationship adjustment information, we had information on *CD38* for 118 individuals. As hypothesized, variation in rs3796863, a single nucleotide polymorphism (SNP) identified in prior work, predicted communal behaviors (e.g., the expression of affection), as well as overall relationship adjustment, such that individuals with the CC (vs. AC/AA) allele reported higher levels of communal behavior across their daily interactions with their romantic partner, as well as higher levels of relationship adjustment. Individuals with the CC (vs. AC/AA) allele of rs3796863 also reported less negative affect and felt insecurity in their interactions with their romantic partner. Notably, we found that variation in the romantic partner's rs3796863 SNP was related to the person's outcomes, independent of the person’s rs3796863 genotype. These findings support the role of oxytocin in the interpersonal processes implicated in the maintenance of close relationships.

Close relationships are essential to psychological and physical health^1–4^. Social connection not only promotes well-being but is related to mortality; simply stated, individuals with fewer social ties are more likely to die^1,2^. Any relationship characterized by frequent interactions and persistent caring should be capable of satisfying our “need to belong”^5^, but romantic relationships may be especially influential as they are the most salient close relationship for most adults^6^. Given the importance of close relationships for survival, it is thought that biological mechanisms evolved to support their formation and maintenance^5,7^. However, the specific systems involved, and how they relate to the higher order psychological processes that characterize close romantic relationship dynamics are not well understood.

One widely recognized candidate is the neuropeptide oxytocin. Decades of research, mainly from rodents and especially the socially monogamous prairie vole indicates that oxytocin influences a range of cognitive and behavioral processes that support attachment in general^8–15^, and romantic bonding specifically^8,10,11,14^. Although research on oxytocin and human attachment has been slower to come about, largely because of methodological limitations (e.g.,^16^), there is some evidence that oxytocin plays a role in romantic bonding in humans as well. For example, oxytocin, measured in blood plasma, predicted frequency of physical contact^17^ and perceived support^18^ in romantic couples. Plasma and/or salivary oxytocin also predicted relationship quality in married couples^19^, as well as relationship survival in dating couples^20^ and parents going through the perinatal transition^21^. Other work suggests that peripheral oxytocin is related to specific behaviors that promote relationship well-being—for example, oxytocin levels were linked to acceptance and self-disclosure during a social support task in couples^22^, and feeling more understood and loved by a romantic partner when expressing and receiving gratitude^23^. Pharmacological studies also support role of oxytocin in processes that promote relationship well-being. In one study of romantic couples, participants who received intranasal oxytocin (vs. placebo) displayed more positive communication behaviors, including more eye contact and emotional self-disclosure, and were less defensive and belligerent during a conflict discussion^24^. In other research, intranasal oxytocin was shown to augment a more “communal” orientation, especially in those who tend to be low in communion; specifically, participants described themselves as more “kind,” “warm,” “gentle,” “caring,” “devoted,” “understanding,” and “emotional” when they received oxytocin, compared to when those same participants received placebo^25^.

Finally, there is also evidence from behavioural genetic studies supporting the role of oxytocin in human attachment and bonding. To date, the oxytocin receptor gene, *OXTR,* has received the most research attention. Although there is evidence linking *OXTR* variation with human social cognition and behaviour (e.g.,^26^), findings have been mixed (see, e.g.,^27,28^). Moreover, *OXTR* does not appear to play an important role in romantic relationships^29^. The other gene of interest is *CD38*, a transmembrane glucoprotein implicated in various physiological processes^30,31^. In a pioneering study, Jin and colleagues^30^ created mice with a null mutation in *CD38*. Compared to wild-type mice, male and female *CD38* knockout mice exhibited profound deficits in social memory and maternal care, respectively; notably, these deficits, were associated with *CD38*-dependent decrease in plasma and cerebrospinal fluid oxytocin levels, supporting the role of *CD38* in oxytocin-mediated social cognition and behavior^30^. Preliminary evidence suggests that *CD38* may also be associated with attachment and bonding in humans. For example, compared to individuals homozygous for the A allele, individuals homozygous for the C allele of the *CD38* single nucleotide polymorphism (SNP), rs3796863, reported less alienation from parents and peers and less suicidal ideation^32^, more supportive peer interactions (^33^, but see^34^), and less social anxiety and depression following the experience of chronic interpersonal stress^35^. This *CD38* SNP has also been linked to parenting behavior: individuals homozygous for the C allele (vs. AA) touched their infant less frequently (^36^; note, this study diverges with the other studies noted as *greater* touch is thought to be indicative of more optimal parenting.) Finally, of particular relevance to the present investigation, Algoe and Way^37^ investigated the role of *CD38* in romantic couples. They found that individuals homozygous for the C allele of rs3796863, as well as another *CD38* SNP, rs6449182, were more likely to express gratitude—an experience that promotes bonding and mutual responsiveness (e.g.,^38^)—during a lab-based interaction and in daily life. Moreover, a composite variable based on both SNPs predicted relationship satisfaction, perceived partner responsiveness, and positive emotions, particularly love, after the lab-based interaction. Although the precise mechanism by which this SNP influences human sociality has not been established, it is thought that these effects reflect variation in the functioning of the oxytocin system given the aforementioned research by Jin et al.

Taken together, research assaying peripheral oxytocin levels, pharmacological challenge studies, and genetic studies, suggest that oxytocin may very well play a role in romantic bonding in humans. One limitation of this prior work, however, is that the majority of these studies focused on single-occasion assessments, often within the confines of the laboratory. Although laboratory studies offer a high degree of experimental control, they are limited in the kinds of interpersonal situations (e.g., conflictual) that can convincingly be recreated, as well as in the number of situations in which participants are willing to be observed. There are also limitations in the range of behaviors and emotions that can be evoked in the laboratory. Methods that study interpersonal dynamics in daily life can circumvent some of these limitations and allow researchers to examine the generalizability of laboratory-based research findings. Moreover, by allowing researchers to obtain multiple measures of an individual’s behaviour in the natural setting of daily life, they yield more reliable estimates of a person’s typical, trait-like behaviour than do studies based on single-occasion assessments.

Here, we aimed to examine the role of oxytocin in romantic relationship dynamics as they unfold in daily life. To this end, we drew upon an existing sample of romantic couples from the community who had participated in a study using an event-contingent recording (ECR) methodology to assess participants’ interpersonal behavior, perception, and affect in their daily social interactions^39^. In this prior study, couples were instructed to complete a standardized ECR form immediately after their significant social interactions (i.e., those involving at least 5-min of synchronous exchanges) over a 20-day period. The ECR form enlists statements sampling different kinds of interpersonal behaviors enacted with high frequency during daily social interactions^40^. These behaviors represent the two dimensions of the interpersonal circumplex^41–43^: the communal dimension ranging from cold/quarrelsome behavior (e.g., “I made a sarcastic comment”) to warm/agreeable behavior (e.g., “I expressed affection with words or gestures”); and the agentic dimension ranging from submissive behavior (“I did not express disagreement when I thought of it”) to dominant behavior (“I asked the other to do something”). Participants were instructed to indicate which behaviors they engaged in during the interaction. Participants also indicated their perceptions of their partner’s behavior along the dimensions of communion and agency, and reported on their affective experience, and how secure/insecure they felt during the interaction; they also completed several measures of relationship adjustment (see "Original study measures").

There are several strengths of the ECR methodology and this dataset in particular. First, participants report on their interpersonal behavior, perception, and affect in a range of situations, which vary along objective (e.g., time of day and place) and subjective/psychological (e.g., degree of conflict vs. affiliation) dimensions; as noted, in addition to enhancing ecological validity, aggregation increases measurement reliability and, in turn, statistical power (see, e.g.,^44^). Second, the ECR methodology minimizes biases in reporting due to memory recall^45^. Because the ECR procedure requires participants to report immediately following a social interaction, these measures are less influenced by various cognitive and motivational processes that may operate at the time of recall^45^; it can therefore be argued that the ECR measures represent with greater veracity the individual’s ongoing and contemporaneous behavior, perception, and affect in a social interaction. Third, because each member of the couple completed the ECR procedure, it is possible to examine both the “actor” and “partner” effects—that is, how the person’s, or actor’s, *CD38* genotype is related to the person’s thought, affect and/or behavior as well as how the person’s *CD38* genotype relates to the partner’s relational experience. Such an examination is especially important in romantic relationships because of the strong interdependence that characterizes the outcomes of both members of a couple. To our knowledge, very few studies investigating genetic correlates of human behaviour have linked a person’s genotype to *their partner’s* experience. Fourth, the ECR measures, when combined with relationship adjustment measures, permit us to examine convergent evidence for the association between *CD38* and ECR measures of behavior, perception, and affect. The overall quality of one’s relationship should reflect the quality of daily interactions; with the present dataset we can test this assumption.

Following Algoe and Way^37^, we focused our analyses on the *CD38* SNP rs3796863. We did not include rs6449182 in our examination due to restricted variability (only one participant was found to be homozygous for the G allele). Our primary hypotheses concerned communal behaviour and global relationship adjustment, given the aforementioned research linking oxytocin with communal behavior^25^, and overall relationship quality^19,21–24^. Specifically, we hypothesized that individuals homozygous for the C allele (vs. individuals who were carriers of the A allele) would report higher levels of communal behavior during their daily interactions with their romantic partner, and higher levels of relationship adjustment. If *CD38* is associated with communal behavior, and if more adjusted dyads are those in which partners engage in more communal behavior, then *CD38* should also be associated with relationship adjustment. As noted, the ECR forms also assess affect and felt security; although these variables were not primary outcomes, we included them in our analyses as secondary outcomes as we thought these indicators of subjective experience would be affected by communion and reflected in relationship adjustment. Finally, we also tested the association between rs3796863 and agentic behavior as this would allow us to examine the specificity of the effects of rs3796863 on communion.

Data were analyzed using the Actor-Partner Interdependence Model (APIM)^46^, which is well suited to analyze dyadic data characterized by the non-independence of observations. As noted, an actor’s outcomes can be influenced not only by their own characteristics but also by their partner’s characteristics. Thus, for each dependent variable, we examined simultaneously the “actor” or “within-partner” associations (the actor’s rs3796863 genotype and, e.g., the actor’s communal behavior) and the “partner” or “cross-partner” associations (the actor’s rs3796863 genotype and, e.g., the partner’s communal behavior). Although we had no a priori hypotheses about gender differences, we tested whether the “actor” and “partner” associations differed between men and women. Finally, we probed the relative contributions of actor and partner effects; specifically, testing for the presence of an actor-only pattern, partner-only pattern, or couple pattern (i.e., equal contribution of actor and partner variables on the actor’s outcomes; see "Data analytical strategy for present investigation").

The original sample consisted of 131 couples. Participants who completed the ECR procedure (i.e., 92 couples; 184 individuals) reported an average of 55.44 interactions with their partner (see "Materials and methods"). Of these 131 couples, we were able to successfully genotype 118 individuals. To maximize the sample of participants, however, we used full maximum likelihood estimator (FIML) with robust standard errors (MLR), which allowed us to make use of the complete dataset from couples reporting ECR and relationship adjustment measures^47,48^ (see "Participants" and "Data analytical strategy for present investigation"). Because we drew upon an existing dataset, we could not conduct a priori power analyses. To address statistical power, we conducted two post-hoc power analysis, one using Monte Carlo simulations in Mplus (see^49^), and the other using a tool designed to estimate power in actor-partner interdependence models^50^. Results from both of these analyses indicate that our study was adequately powered to detect small to medium size associations expected between *CD38* rs3796863 and our key outcome variables (see "Power analysis").

## Results

We first summarize results for our primary outcomes: communal behavior and global relationship adjustment. We then sumarize results for our secondary outcomes: perceived communal behavior in partner, negative and positive affect, and felt-insecurity/security. We conclude with exploratory outcomes: agentic behavior and perceived agency in partner. For each dependent variable, we tested gender differences in the within- and cross-partner effects, and then whether there was evidence of a couple pattern. For ease of presentation, we only describe the significant effects from the analyses below. Unless a gender difference was found, the subsequently presented findings represent pooled effects across both genders. The main findings are presented in Tables 1, 2 and 3, and a descriptive summary of the results can be found in Table 4. A complete presentation of the findings can be found in Tables S1 and S2 in Online Supporting Information (OSI), along with the within- and cross-partner correlations among the study variables (Table S3). Finally, as noted, we had missing genetic information from some participants; although our data analytic approach allowed us to use the data from the original couples in which both members successfully completed the ECR procedure (92 couples), and the relationship quality measures (111 couples), we also adopted a more conservative approach and ran our analyses using only participants for whom we had genetic information (both members of 39 couples and 1 member from 26 couples for ECR variables, and both members of 43 couples and 1 member from 31 couples for relationship adjustment). Critically, the main results from those analyses were similar to the ones presented below (see OSI Tables S9 and S10).

**Table 1:** Associations between the actor’s and partner’s CD38 rs3796863 genotype and communal behavior, agentic behavior, affect, perception of the partner’s communal and agentic behavior, and feelings of security with the romantic partner.

	χ^2^	Unstnd Estimate (SE)	Stnd. Estimate	z	*p*	95% CI
**Actor’ rs3796863 → Actor’s**						
Com. Beh.	.67	**− .069 (.024)**	**− .280**	**− 2.859**	**.004**	**− .116, − .022**
Perc. Com.	.69	**− .386 (.164)**	**− .191**	**− 2.360**	**.018**	**− .707, − .065**
Neg. Affect	2.63	**.250 (.090)**	**.272**	**2.761**	**.006**	**.072, .398**
Pos. Affect	1.27	**− .325 (.158)**	**− .176**	**− 2.058**	**.040**	**− .635, − .015**
Felt Insec.	1.26	**.220 (.099)**	**.230**	**2.208**	**.027**	**.025, .414**
Felt Sec.	1.33	**− **.187 (.177)	**− **.092	**− **1.062	.288	**− **.533, .159
Age. Beh.	4.62*					
Women		.033 (.03)	.173	1.227	.220	**− **.020, .086
Men		**− **.041 (.02)	**− **.247	**− **1.743	.081	**− **.088, .005
Perc. Age.	1.30	.243 (.258)	.111	0.942	.346	**− **.263, .748
**Partner’ rs3796863 → Actor’s**						
Com. Beh.	2.05	**− .055 (.023)**	**− .222**	**− 2.404**	**.016**	**− .099, − .010**
Perc. Com.	.19	**− **.249 (.174)	**− **.124	**− **1.433	.152	**− **.590, .092
Neg. Affect	1.17	**.190 (.084)**	**.207**	**2.626**	**.024**	**.025, .329**
Pos. Affect	**2.92**^**†**^					
Women		**− **.344 (.312)	**− **.157	**− **1.101	.271	**− **.955, .268
Men		.179 (.179)	.112	.999	.318	**− **.172, .529
Felt Insec.	.32	.141 (.108)	.148	1.310	.190	**− **.070, .353
Felt Sec.	2.38	**− **.060 (.173)	**− **.030	**− **0.349	.727	**− **.400, .279
Age. Beh.	9.38**					
Women		.019 (.03)	.097	.697	.486	**− **.034, .072
Men		**− .069 (.02)**	**− .418**	**− 2.940**	**.003**	**− .115, − .023**
Perc. Age.	.67	.139 (.205)	.064	0.680	.497	**− **.262, .541

*N* = 92 couples (184 participants), between 7,450 and 7,579 daily events.

“Com. Beh.”, Communal Behavior; “Perc. Com.”, Perceived communal behavior; “Neg. Affect”, Negative affect; “Pos. Affect”, Positive affect; “Felt Insec.”, Felt insecurity; “Felt Sec.”, Felt security; “Age. Beh.”, Agentic behavior; “Perc. Age.”, Perceived agentic behavior. The χ^2^ indicates the difference in fit of a model in which the estimate was permitted to differ between genders with the fit of a model in which the estimate was restricted to be equal in both genders; a nonsignificant χ^2^ indicates the absence of a gender difference. Significant effects are in bold.

†*p* < .10; *p < .05; **p < .01; ***p < .001. The presented estimates are those pooled across men and women (i.e., when no gender difference was found). For the gender-specific estimates obtained in the unrestricted APIM models (i.e., when estimates were free to differ across gender), refer to the estimates presented in the “Women” and “Men” columns in Table S1 in OSI. In Table S1, the estimates in italics in the same “Women” and “Men” columns are those found to be statistically different between genders. “Unstnd” = Unstandardized; “Stnd” = Standardized; “CI” = Confidence interval.

**Table 2:** “Couple Pattern” associations between CD38 rs3796863 genotype and communal behavior, agentic behavior, affect, perception of the partner’s communal and agentic behavior, and feelings of security with the romantic partner.

Couple pattern?	χ^2^	Unstnd. Est. (SE)	Stnd. Estimate	z	*p*	95% CI
Com. Beh.	.28	**− .062 (.019)**	**− .250**	**− 3.196**	**.001**	**− .099, − .024**
Perc. Com.	.74	**− .322 (.147)**	**− .160**	**− 2.191**	**.028**	**− .611, − .034**
Neg. Affect	.53	**.190 (.077)**	**.241**	**2.877**	**.004**	**.070, .371**
Pos. Affect						
Women	.00	**− .329 (.157)**	**− .152**	**− 2.099**	**.036**	**− .636, − .022**
Men	7.22**					
Felt Insec.	1.04	**.181 (.095)**	**.190**	**1.903**	**.057**	**− .005, .368**
Felt Sec.	.69	**− **.117 (.158)	**− **.058	**− **0.743	.457	**− **.427, .192
Age. Beh.						
Women	.56	.027 (.021)	.141	1.250	.211	**− **.015, .068
Men	.18	**− .057 (.015)**	**− .342**	**− 3.824**	**.000**	**− .086, − .028**
Perc. Age.	.15	.180 (.183)	.082	0.981	.326	**− **.179, .539

*N* = 92 couples (184 participants), between 7,450 and 7,579 daily events.

“Com. Beh.”, Communal behavior; “Perc. Com.”, Perceived communal behavior; “Neg. Affect”, Negative affect; “Pos. Affect”, Positive affect; “Felt Insec.”, Felt insecurity; “Felt Sec.”, Felt security; “Age. Beh.”, Agentic behavior; “Perc. Age.”, Perceived agentic behavior. The χ^2^ value indicates the difference in fit of a model in which the “actor” and “partner” estimates were permitted to differ with the fit of a model in which these estimates were restricted to be equal; a nonsignificant χ^2^ indicates the presence of a couple pattern. For men’s positive affect value, refer to actor and partner values in Table 1. Significant effects are in bold. **†p** < .10; *p < .05; **p < .01; ***p < .001. The presented estimates are those pooled across actor and partner estimates (i.e., when a couple pattern was present). “Unstnd” = Unstandardized; “Stnd” = Standardized; “CI” = Confidence interval.

**Table 3:** Associations between the actor’s and partner’s CD38 rs3796863 genotype and relationship adjustment.

	χ^2^	Unstnd Estimate (SE)	Stnd. Estimate	z	*p*	95% CI
**Actor’ rs3796863 → Actor’s:**						
Rel. Adjust.	1.24	**− .633 (.207)**	**− .292**	**− 3.058**	**.002**	**− 1.039, − .227**
**Partner’ rs3796863 → Actor’s:**						
Rel. Adjust.	1.02	**− .426 (.226)**	**− .199**	**− 1.886**	**.059**	**− .869, .017**

*N* = 111 couples (222 participants). “Rel. Adjust.”, Relationship adjustment. Model fit indices for the Relationship Adjustment Model: (1) estimates free to vary across genders: χ^2^ (155, N = 111) = 237.53, p = .001; CFI = .908; RMSEA = .069; SRMR = .121; (2) gender equality established: χ^2^ (157, N = 111) = 238.61, *p* = .001; CFI = .909; RMSEA = .068; SRMR = .118; and (3) couple pattern established: χ^2^ (158, N = 111) = 240.21, *p* = .001; CFI/TFI = .908; RMSEA = .068; SRMR = .119. The χ^2^ indicates the difference in fit of a model in which the estimate was permitted to differ between genders with the fit of a model in which the estimate was restricted to be equal in both genders; a nonsignificant χ^2^ indicates the absence of a gender difference. Significant effects are in bold. †*p* < .10; *p < .05; **p < .01; ***p < .001. The presented estimates are those pooled across men and women (i.e., when no gender difference was found). For the gender-specific estimated obtained in the unrestricted APIM models (i.e., when estimates were free to differ across gender), refer to the estimates presented in the “Women” and “Men” columns in Table S2 in OSI. “Unstnd” = Unstandardized; “Stnd” = Standardized; “CI” = Confidence interval.

**Table 4:** Summary of findings about CD38 rs3796863 and ECR and relationship adjustment measures.

	Effect	Within-partner effect?	Cross-partner effect?	Couple-pattern?
**Primary outcomes**				
Communal behavior	CC > AA/AC	**Yes**	**Yes**	**Yes**
Relationship adjustment	CC > AA/AC	**Yes**	**Yes**†	**Yes**
**Secondary outcomes**				
Perceived partner communal behavior	CC > AA/AC	**Yes**	No	**Yes**
Negative affect	CC < AA/AC	**Yes**	**Yes**†	**Yes**
Positive affect	CC > AA/AC	**Yes**	No	**Yes** (effect only in women)
Felt insecurity	CC < AA/AC	**Yes**	No	**Yes**†
Felt security	CC > AA/AC	No	No	No
**Exploratory outcomes**				
Agentic behavior	CC > AA/AC	No	**Yes** (effect only in men)	**Yes** (effect only in men)
Perceived partner agentic behavior	CC > AA/AC	No	No	No

Couple pattern established when there are no significant differences in the within-partner and cross-partner effects; that is, the contributions are equal. Significant effects are in bold. †*p* < .10.

### Primary outcomes

#### Communal behavior

**Figure 1:**
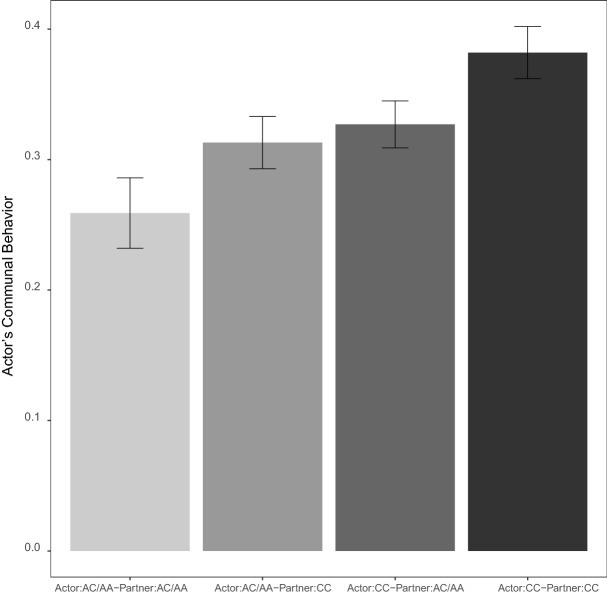
Bar graph illustrating the additive effects of actor’s and partner’s *CD38* rs3796863 genotype on actor’s communal behavior. Communal behavior was highest among actor’s with the CC genotype whose partner was a carrier of the CC genotype. *n* = 7,579 observations from 184 participants. Error bars represent ± 1 SEM.

Results revealed significant within- and cross-partner effects of rs3796863: Actors with the CC genotype reported engaging in more communal behavior across all daily interactions with their partners than actors with the AC/AA genotype. The cross-partner association between rs3796863 and communal behavior was also significant: Actors whose partner had the CC genotype also reported engaging in more communal behavior than actors whose partner had the AC/AA genotype (Table 1). A couple pattern was observed such that no statistical difference in the within- and cross-partner associations was found: χ^2^_(1) _= 0.28, *p* = 0.597 (Table 2). When pooled across within- and cross-partner effects, the association between rs3796863 and communal behavior was significant. As depicted in Fig. 1, actors reported engaging in more communal behavior when they and their partner had the CC genotype (estimate = 0.38, *SE* = 0.02,* z* = 18.80, *p* < 0.001) than when (a) one of them had one or both copies of the A allele (estimate = 0.32, *SE* = 0.01,* z* = 23.54, *p* < 0.001) or (b) both of them had the AC/AA genotype (estimate = 0.26, *SE* = 0.03,* z* = 9.84, *p* < 0.001).

#### Relationship adjustment

Results revealed significant within-partner effects of rs3796863: Actors with the CC genotype reported higher adjustment than actors with the AC/AA genotype. The cross-partner association between rs3796863 and relationship adjustment was marginally significant (Table 3). Analyses support a couple pattern as the within- and cross-partner associations were not statistically different: χ^2^_(1)  _= 1.61, *p* = 0.205. The pooled association was significant, *b* =  − 0.545, *SE* = 0.200,* z* =  − 2.717, *p* = 0.007. Actors’ reported higher relationship adjustment when they and their partner had the CC genotype (estimate = 8.24, *SE* = 0.74,* z* = 11.16, *p* < 0.001) than when (a) one of them had one or both copies of the A allele (estimate = 7.69, *SE* = 0.62,* z* = 12.37, *p* < 0.001) or (b) both of them had the AC/AA genotype (estimate = 7.15, *SE* = 0.56,* z* = 12.86, *p* < 0.001).

### Secondary outcomes

#### Perception of partner’s communal behavior

Results revealed a significant within-partner effect of rs3796863: Actors with the CC genotype perceived greater communal behavior overall in the partner than actors with the AC/AA genotype (Table 1). Results also indicated the presence of a couple pattern (Table 2). Pooled across within- and cross-partner associations, the association between rs3796863 and perceived communal behavior was significant. Actors perceived greater communal behavior when both the actor and partner had the CC genotype (estimate = 8.93, *SE* = 0.18,* z* = 50.73, *p* < 0.001) than when (a) one of them was a carrier of one or both copies of the A allele (estimate = 8.61, *SE* = 0.10,* z* = 83.07, *p* < 0.001) or (b) both of them had the AC/AA genotype (estimate = 8.29, *SE* = 0.18,* z* = 45.09, *p* < 0.001).

#### Negative affect

Results revealed a significant within-partner effect of rs3796863: Actors with the CC genotype experienced less negative affect across their daily interactions with their romantic partner than actors with the AC/AA genotype; the cross-partner effect fell short of conventional levels for statistical significance (Table 1). As detailed in Table 2, results indicate the presence of a couple pattern: Actors experienced less negative affect when both the actor and partner had the CC genotype (estimate = 0.34, *SE* = 0.08,* z* = 4.24, *p* < 0.001) than when (a) one of them was a carrier of one or both copies of the A allele (estimate = 0.56, *SE* = 0.04,* z* = 12.86, *p* < 0.001) or (b) both of them had the AC/AA genotype (estimate = 0.78, *SE* = 0.10,* z* = 8.10, *p* < 0.001).

#### Positive affect

Results revealed a significant within-partner effect of rs3796863: Actors with the CC genotype experienced more positive affect than actors with an AC/AA genotype. The cross-partner association between rs3796863 and positive affect was not significant (Table 1). Results revealed a couple pattern only in women (see Table 2): Female actors reported experiencing more positive affect when they and their partner had the CC genotype (estimate = 3.39, *SE* = 0.13,* z* = 25.41, *p* < 0.001) than when (a) one of them was a carrier of one or both copies of the A allele (estimate = 3.06, *SE* = 0.134,* z* = 22.80, *p* < 0.001) or (b) both of them had the AC/AA genotype (estimate = 2.73, *SE* = 0.26,* z* = 10.52, *p* < 0.001). Conversely, positive affect for men was only related to their own genotype (“actor” effect =  − 0.329, *SE* = 0.157,* z* =  − 2.099, *p* = 0.036), not their partner’s genotype (“partner” effect = 0.176, *SE* = 0.176,* z* = 1.002, *p* = 0.316).

#### Felt insecurity

Results revealed a significant within-partner effect of rs3796863: Actors with the CC genotype felt less insecure across their interactions with their romantic partner than actors with an AC/AA genotype (Table 1). Results indicate the presence of a couple pattern (Table 2), although the pooled effect estimate fell short of statistical significance (*p* = 0.057). Actors felt less insecure during their interactions when both they and their partner had the CC genotype (estimate = 0.40, *SE* = 0.11,* z* = 3.62, *p* < 0.001) than when (a) one of them was a carrier of one or both copies of the A allele (estimate = 0.58, *SE* = 0.05,* z* = 11.47, *p* < 0.001) or (b) both of them had the AC/AA genotype (estimate = 0.76, *SE* = 0.11,* z* = 7.27, *p* < 0.001).

#### Felt security

Results showed no significant within- or cross-partner associations of rs3796863 (Table 1); neither was there evidence for a couple pattern (Table 2).

### Exploratory outcomes

#### Agentic behavior

Results showed no significant within-partner association between rs3796863 and agentic behavior (Table 1), indicating that variation in rs3796863 appears to be specifically related to an actor’s communal behavior.

Unexpectedly, results revealed a significant cross-partner effect for men: male actors whose partner had the CC genotype reported engaging in more agentic behavior than male actors whose partner had the AC/AA genotype. A couple pattern was also observed for men (Table 2). Male actors reported engaging in more agentic behavior when they and their partner had the CC genotype (estimate = 0.17, SE = 0.02, z = 9.19, *p* < 0.001) than when (a) one of them was a carrier of one or both copies of the A allele (estimate = 0.12, SE = 0.01, z = 12.01, *p* < 0.001) or (b) both of them had the AC/AA genotype (estimate = 0.06, SE = 0.02, z = 3.85, *p* < 0.001). While a couple-pattern was also found for women (Table 2), the pooled within- and cross-partner effect was not significant. Thus, while variation in actors’ rs3796863 did not appear to influence the actors’ behavior, having a female partner who possessed the CC genotype of rs3796863 was associated with more agentic behavior in male actors.

#### Perception of partner’s agentic behavior

Results showed no significant within- or cross-partner associations for rs3796863 (Table 1); neither was there evidence for a couple pattern (Table 2).

## Discussion

We demonstrate, for the first time, that *CD38*, a gene linked to the oxytocin secretion and social behavior in rodents^30^, is also involved in regulating communal/affiliative behavior in human romantic relationship dynamics as they unfold in daily life. Specifically, we show that *CD38* rs3796863 is associated with an individual’s communal behavior, such as the expression of affection in daily interactions with a romantic partner: individuals who were homozygous for the C allele (i.e., CC) reported engaging in more communal behavior than individuals who were carriers of the A allele. *CD38* rs3796863 was also associated with perceptions of one’s partner’s communal behavior; those with the CC genotype (vs. AA/AC) were more likely to see their partner as behaving more communally across their social interactions. Of note, we did not observe any association between rs3796863 and agency (behavior or partner perceptions), which highlights the specificity of the association between rs3796863 and communion, consistent with theory and research linking oxytocin specifically to care-giving behavior (e.g.,^12,25,51^). Not only was *CD38* rs3796863 associated with communal behavior and perception of communion in the partner, it was also related to individuals’ subjective experience during their interactions with the partner, with those with the CC genotype experiencing less negative and more positive affect, as well as less felt insecurity. Finally, *CD38* rs3796863 was associated with relationship adjustment: those with the CC genotype reported higher levels of global relationship adjustment than individuals with the AA/AC genotypes. This is notable given that our measure of global relationship adjustment assessed multiple facets of the relationship including perceptions of relationship quality, personal need satisfaction in the relationship, and perceptions of support from the partner, thereby providing a comprehensive assessment of relationship quality.

To our knowledge, this is one of the few studies in the field of human behavioral genetics to adopt a multi-method approach to obtain contemporaneous measures of an individual’s behavior, perception, and affect in daily social interactions, as well as global measures of adjustment in close relationships. To the extent that the quality of a relationship depends on the quality of daily interactions—as it theoretically should—the ECR measures can be conceived as indicators of interpersonal processes that give rise to higher order psychological phenomena such as an individual’s evaluation of his/her relationship. For example, how communal individuals behave and/or how much negative affect they experience with their partner should shape their assessment of relationship quality^52^. The present findings thus provide converging evidence regarding the involvement of *CD38* in regulating psychological processes that support human romantic relationships.

Additional converging evidence for the role of *CD38* in romantic relationship dynamics comes from the “partner” effects—that is, whether and how the participant’s partner’s *CD38* rs3796863 genotype is associated with the participant’s behavior, perception, and affect. In both men and women, we observed a couple pattern; that is, participants’ communal behavior, their perception of their partner’s communal behavior, as well as their experience of negative affect, and relationship adjustment, were equally related to their partner’s rs3796863 genotype as to their own rs3796863 genotype. In other words, a participant’s standing on each of these variables depended as much on their own genotype as on their partner’s genotype. Indeed, we observed the highest levels of communal behavior, and of perceptions of the partner’s communal behavior, when both the participant and the partner possessed the CC genotype; conversely, we observed the lowest levels of communal behavior and of perceptions of the partner’s communal behavior when neither partner possessed the CC genotype. These findings indicate that having a partner with a CC genotype can compensate for the lower levels of communal behaviour, and perceived communal behaviour, manifested by participants with the AA/AC genotypes. So long as their partner carried the CC genotype, participants with the AA/AC genotype reported comparable levels of communal behavior as participants with the CC genotype. A similar pattern of findings was observed for negative affect, and global relationship adjustment. Given these partner effects, one may speculate that individuals with certain genotypes select partners that will compensate for their deficits; however, we did not find evidence of assortative mating in this study as indicated by a non-significant correlation between the two partners’ rs3796863 genotype (see "Genetic study procedures and genotyping").

The relevance of this couple pattern demonstrating converging evidence for the involvement of *CD38* in romantic relationship dynamics is of considerable theoretical importance. If *CD38* is involved in regulating communal behavior in the partner, and if the partner’s communal behavior evokes/elicits communal behavior from the participant^41,42^, then it follows that the partner’s *CD38* genotype should be associated with the participant’s communal behavior, which is what we found. Furthermore, since communal acts are thought to affirm connection, closeness with, and love for the other^40^, a partner’s communal behavior should have downstream effects on the participant’s perception of communal behavior, affect, feelings of security, and, over time, judgments about relationship quality. These interpersonal dynamics, we suggest, may account for our findings linking a partner’s *CD38* rs3796863 genotype and the participant’s perceived partner communion and negative affect during daily interactions, and participant’s global relationship adjustment. Over time, these perceptual and affective experiences may give rise to a self-sustaining and reinforcing cycle of reciprocated communal behavior between romantic partners; in this way, these findings shed light on how oxytocin via its effects on communion may support bonding in close romantic relationships.

Our finding showing that individuals with the rs3796863 CC genotype perceived higher levels of communal behavior in their partner conceptually replicates those reported by Algoe and Way^37^, who found that individuals with the *CD38* rs3796863 CC genotype were more likely to see their romantic partner as more responsive following their expression of gratitude. Our findings are also consistent with other evidence suggesting that individuals who were homozygous for the C allele of rs3796863 reported feeling less alienated from parents and peers^32^, more supported in their social interactions^33^, and less anxiety following the experience of chronic interpersonal stress^35^. That said, our findings contrast with those observed by Feldman et al.^36^, who found that individuals with the rs3796863 CC genotype displayed less optimal parenting behaviors (less parental touch and shorter durations of parent-infant gaze synchrony). The discrepancy, however, may be due to differences in the social context in which those behaviors were studied, namely, adult-adult verus parent–child relationships. Population differences related to ethnicity and/or socio-cultural factors, which have been shown to moderate the effects of oxytocin-related genes (e.g.,^53^), may also play a role since our sample consisted of North Americans, whereas Feldman’s^35^ sample was of Israeli-Jewish ethnicity. It will be important for future work to address the boundary conditions for the effects we report.

One question raised by this research concerns the precise relationship between variation in *CD38* SNPs and the oxytocin system in humans. *CD38* is thought to influence social-affiliative behavior in mice (and, presumably, in humans) via its effects on oxytocin secretion and likely oxytocin release within the brain (cf.^30^). To our knowledge, however, the functional significance of this polymorphism in humans is not known. Indeed, as Algoe and Way^37^ point out, one should be cautious in making direct links between variation in *CD38* SNPs, oxytocin levels and social behavior since polymorphisms of *CD38* are likely only indirect indicators of oxytocin secretion. Future work is needed to understand the functional significance of this polymorphism.

A few comments about our sample are worth noting. First, our sample was ethnically mixed (70% Caucasian); although our analyses indicated that this was not an issue of concern (see "Genetic study procedures and genotyping" and "Materials and methods"), future work is needed to replicate these findings in more ethnically homogenous samples and to examine their generalizability to other ethnicities and cultures. Second, although our data analytic approach allowed us to make use of the complete original dataset, we did have missing genetic information from some participants. That said, the main findings reported here held when we adopted a more conservative approach and restricted our sample to the couples for whom we had genetic information.

Third, our participant sample size was not large (although it was comparable to that reported by Algoe and Way^37^). The ECR procedure is labor intensive and costly so we analyzed existing data for this initial study; therefore, we had little control over sample size. That said, several factors speak to the reliability of the effects we report. First, as noted, the consistency in the pattern of results we observed, using different methods, at different levels of analysis (i.e., perception of overall relationship adjustment vs. perception of discrete interpersonal interactions), increases our confidence that these findings are not spurious. Second, our within-subject sample size was large: in contrast to single-occasion assessments we had, on average, 55 event-level observations of participants’ interpersonal behavior, perception, and affect. It is well-established that aggregating across measures increases measurement reliability and, in turn, increases statistical power and decreases the probability of type II error (see OSI for a discussion about statistical power in the context of multilevel analysis and how the event level sample size contributes to statistical power in this study). Third, both of our post-hoc power analyses indicate that our study was adequately powered to detect small to medium size associations between *CD38* rs3796863 and our key outcome variables. Forth, it is worth reiterating that this is a conceptual replication and extension of prior work, as opposed to an entirely novel finding based on a-theoretical explorations. In sum, we believe our use of the ECR procedure offsets some of the limitations of our sample size. Increasing participant sample size is one way to bolster confidence in the reproducibility of an effect, but so too is using intensive measurement methods that assess individuals on multiple occasions, across a range of naturally occurring situations. Of course, as with any research, future replication studies will be important.

In conclusion, abundant evidence from rodent studies indicates that the oxytocin system plays a key role in pair-bonding, and recent work assaying peripheral oxytocin and examining the effects of intranasal oxytocin administration, as well as genetic studies, suggests that oxytocin is also involved in regulating romantic relationships in humans. In line with this work, we show that variation in *CD38* plays a key role in the communal-affiliative behaviors that support bonding in humans. A basic supposition of the research on the neurobiology of attachment is that humans evolved to have a proclivity for forming enduring pair-bonds; *CD38* may be an important factor in this process. Implicit in our work is the notion that these communal affiliative behaviors facilitate relationship longevity, but future work is needed to directly assess the effects of *CD38* on romantic relationship survival.

## Materials and methods

### Participants

We drew upon an existing dataset of 131 heterosexual couples for the present investigation (see "Original ECR study details", below, for details on that study and additional participant information). Specifically, four years after that study was completed, we contacted participants and invited them to participate in a study examining genetic correlates of social behavior. One hundred and forty-five participants (55% of the original sample) responded to this invitation; 11 declined to participate, 10 agreed to participate but failed to complete the study procedures, and two participants did not return their study materials in a timely fashion. Of the final 122 individuals, there were four participants for whom the genetic data could not be analyzed; there were thus 118 participants (both members of 43 couples and one member from 32 couples) for whom we had genetic information. The original sample, being heterosexual couples, had an equal number of men and women; the participants for whom we had genetic information consisted of 65 women and 53 men. The sample was primarily Caucasian (70.34%) and varied in educational background; average relationships length was 58.19 ± 69.58 months (range = 8–384 months; see OSI Table S4 for a comprehensive description of sample demographics and "Genetic study procedures and genotyping" for details regarding genotype distribution).

Although we only had genetic information from 118 participants, with our data analytic approach we were able to make use of the full dataset; specifically, we included participants’ data in the analyses as long as we had information from both partners on the dependent variable (i.e., ECR measures and relationship adjustment measures), and we allowed participants to have missing genetic information (see "Data analytical strategy for present investigation"). Note that one of the 118 participants for whom we had genetic information did not have ECR or relationship adjustment information so could not be included in the analyses.

Participants who provided a saliva sample (vs. those who did not) did not differ in age (*b* = 0.06, *SE* = 0.04, *z* = 1.44, *p* = 0.15) or relationship length (*b* = 0.06, *SE* = 0.28, *z* = 0.20, *p* = 0.84). Moreover, of those who provided relationship adjustment measures (some couples provided relationship adjustment measures but did not provide usable ECR data so the samples vary slightly), participants who provided a saliva sample were no more likely to be satisfied with their relationship (*b* = 1.10, *SE* = 0.69, *z* = 1.60, *p* = 0.11) as indicated by the total score in the Dyadic Adjustment Scale^54^. That these groups did not differ in measured demographic or relationship characteristics suggests that the pattern of missingness is at random, satisfying the assumption of our data analytic approach.

### Genetic study procedures and genotyping

Participants were contacted and invited to provide a saliva sample and complete a set of questionnaires. Consenting participants were mailed an Oragene self-collection kit (DNA Genotek; www.dnagenotek.com) and a stamped return envelope. At a mutually agreed time, a research assistant (RA) called each participant to assist him/her with data collection. Participants were instructed not to eat, drink, smoke, or chew gum 1 h prior to saliva sample collection; to ensure compliance, they were asked to complete some online questionnaires and, when finished, to phone the RA, who would walk them through the saliva sample collection process. Participants then mailed the completed kit using the envelope provided (this is a standard procedure, see e.g.^55^). Genotyping was performed by Genome Quebec (see OSI for details on genotyping procedures). Participants were compensated CAD $50 for participation. The genetics study was approved by the McGill University Faculty of Medicine Research Ethics Board and was carried out in accordance with the Canadian Tri-Council Policy Statement on Ethical Conduct for Research Involving Humans. Informed consent was obtained from all study participants.

We coded the rs3796863 SNP consistent with prior work, (e.g.,^35–37,56^): {CC genotype = 0 (*N* = 57); A allele carriers [AC (*N* = 52) and AA (*N* = 9)] = 1}. The HardyWeinberg package in R (version 1.5.8;^57^) was used to calculate Hardy–Weinberg Equilibrium values using the chi-square test without the continuity correction. These genotype distributions met Hardy–Weinberg Equilibrium expectations, χ^2^_(1) _= 0.37, *p* = 0.54. Partners’ genotype was not correlated with each other, *r* (Spearman’s rho) = − 0.05,* p* = 0.77.

### Original ECR study details

Participants in the original ECR study were recruited through advertisements in local newspapers, a vendor-organized wedding planning event, and free online classifieds (e.g., Craigslist.ca). Interested couples were invited to participate in a study about social interactions between romantic partners if they had been cohabiting for at least 6 months, had no children living in their household, and held at least a part-time job.

After consenting to participate, participants completed questionnaires and then were introduced to the ECR procedure. Participants were instructed to complete ECR forms following social interactions of at least 5-min duration on each day for the next 20 days. Different kinds of interaction partners (e.g., friend, co-worker) were allowed, but participants were asked to try to report at least 2–3 daily interactions involving their romantic partner (here we focus only on interactions with the partner). Participants were advised to complete the forms in private and to avoid discussing them with their partner. To ensure compliance with the ECR procedure, participants were asked to mail the forms on the day following their completion. The forms were examined upon their arrival to ensure that they were completed correctly and mailed in a timely fashion. Of 131 couples who started the ECR procedure, both members of 92 couples (184 participants or 70% of the recruited sample) complied with the requirement of mailing the completed ECR forms daily. Compliance with the ECR procedure was not associated with age (*p* = 0.17) or relationship length (*p* = 0.38).

At the end of the ECR procedure, participants returned to the lab and completed questionnaires, including relationship adjustment questionnaires, were debriefed, and compensated CAD $160 for their participation. Of the original 131 couples (262 individuals), both members of 111 couples (84% of the recruited sample and 100% of the participants who complied with the ECR procedure) completed the relationship adjustment measures. Compliance with ECR procedure was unrelated to relationship satisfaction, indicating that couples that completed the ECR procedures did not differ from those that did not in terms of relationship satisfaction (*p* = 0.86).

### Original study measures

#### Event-contingent recording (ECR)

On average, the 184 participants who completed the ECR procedure reported 55.44 interactions (*SD* = 18.89, *Range* = 10–127). The 104 participants who provided a saliva sample and completed the ECR procedure reported 55.35 interactions (*SD* = 18.40, *Range* = 10–124).

*Social Behavior Inventory* (SBI;^40^). The SBI was used to measure interpersonal behavior as conceptualized in Interpersonal Circumplex models^41–43^. The SBI consists of 12 items measuring each of the four poles of the interpersonal circle: Agreeable (e.g., “I smiled and laughed with others”); Quarrelsome (e.g., “I made a sarcastic comment”); Dominant (e.g., “I asked the other to do something”); and Submissive (e.g., “I gave in”). Participants were asked to indicate (*yes* = 1; *no* = 0) the behaviors they had engaged in during each interaction. To guard against the possibility of participants adopting a response set, four forms, with different SBI items, were used in a daily rotation; each dimension of behavior was represented by three items on each of the four forms.

Four behavioral scores representing each pole of the interpersonal circle were constructed for each event. First, for each behavioral scale, the number (i.e., frequency: 0 to 3) of items endorsed was calculated. Second, mean scores (frequency/3) were calculated for each behavior scale. Third, to adjust for the participant's general rate of responding, ipsatized scores were constructed by subtracting the mean for all behaviors across all four scales (frequency/12) from each behavior scale mean score. Lastly, event-level communal behavior scores were calculated by subtracting quarrelsome behavior from agreeable behavior; event-level agentic behavior scores were calculated by subtracting submissive behavior from dominant behavior.

*Interpersonal Grid* (IG;^58^). The IG is a single-item instrument consisting of an 11 × 11 matrix of squares arranged along the two axes of agency and communion; the vertical axis represents agency and is anchored by “*assured–dominant*” and “*unassured–submissive*”; the horizontal axis represents communion and is anchored by “*cold–quarrelsome*” and “*warm–agreeable*”. Participants were instructed to place an X in a single square on the grid. Higher scores indicate higher perceived agentic and communal behavior.

*Affect.* Following interpersonal circumplex models of emotion^59,60^, negative affect was measured using five items, *worried/anxious*, *frustrated*, *angry/hostile*, *unhappy*, and *depressed/blue*, and positive affect was measured using four items, *happy*, *pleased*, *enjoyment/fun*, and *joyful*. Items were rated on a scale ranging from 0 (“*not at all*”) to 6 (“*extremely*”). Event-level scores for negative affect and positive affect were constructed for each interaction by computing the mean of the respective item scores, with higher scores indicating higher negative affect and positive affect. McDonald’s omega reliability coefficient^61^ was high for negative and positive affect: 0.93 and 0.95, respectively.

*Felt insecurity/security.* Eight items assessing felt insecurity/security in daily interactions were used^62^. Four items assessed felt insecurity (e.g., “I felt that my partner did not care about my needs”) and four items measured felt security (e.g., “I felt assured about my partner’s feelings/intentions toward me”). Items were rated on a scale ranging from 0 (“*not at all*”) to 6 (“*extremely*”). Omega was high for felt security and felt insecurity scales: 0.96 and 0.90, respectively.

### Relationship adjustment measures

*Dyadic Adjustment Scale* (DAS;^54^). This 32-item measure consists of four subscales measuring facets of relationship adjustment: Affectional Expression (e.g., “Demonstrations of affection”), Satisfaction (e.g., “In general, how often do you think things between you and your partner are going well?”), Consensus (e.g., “Making major decisions”), and Cohesion (e.g., “Do you and your partner engage in outside interests together?”). Subscale scores were calculated by averaging the subscale item scores, with higher scores indicating higher levels of each facet. Omega ranged from moderate to high: Affectional Expression = 0.69; Satisfaction = 0.84; Consensus = 0.83; and Cohesion = 0.77.

*Basic Need Satisfaction in Relationship Scale* (BNSR;^63^). The BNSR consists of 9 items rated from 1 (*not at all true*) to 7 (*very true*), assessing the extent to which participants perceive that their needs for autonomy (e.g., “When I am with my partner, I feel free to be who I am”), competence (e.g., “When I am with my partner, I feel very capable and effective”), and relatedness (e.g., “When I am with my partner, I feel loved and cared about”) are met in their relationship with the partner. Subscale scores were calculated by averaging the subscale item scores, with higher scores reflecting greater satisfaction of each respective need in the relationship. Subscale omegas were moderate: Autonomy = 0.80; Competence = 0.80; Relatedness = 0.76.

*Autonomy Support for Goal Pursuit* (AS;^64^). Five items were rated on a 7-point scale ranging from 1 (*not at all true*) to 7 *(very true*). Autonomy support is defined as support consisting of (1) empathic perspective taking of a person’s needs and (2) feelings and the provision of choices as opposed to the exertion of control and pressure^64,65^. A sample item is “I feel that my partner understands how I see things with respect to my goals.” The mean of item scores was calculated as an index of perceived autonomy support from the partner, with higher scores indicating higher levels of support. In the present sample, omega was moderate: 0.78.

Based on the assumption that the DAS, BNSR, and AS measure facets of relationship adjustment, we aggregated the three relationship adjustment measures, based on a confirmatory factor analysis, to create the higher order latent factor of global relationship adjustment (see OSI Tables S5–S8, for details).

### Data analytical strategy for present investigation

The Actor-Partner Interdependence Model (APIM)^46^ was used to examine associations between rs3796863 genotype and the ECR measures and global relationship adjustment. Analyses were conducted using Mplus 8.1^66^. Multilevel (i.e., two-level) APIM was employed to examine the association between the rs3796863 genotype and ECR measures (e.g., communal behavior). Mplus performs an implicit decomposition of scores from ECR measures into two latent parts: an event-level score (within-person; i.e., the deviation of an event score from the mean across all events) and person-level score (between-person; i.e., mean across all events). The person-level scores were regressed simultaneously on the actor’s and partner’s *CD* rs3796863 genotype. Regarding relationship adjustment, we used one-level APIM (because adjustment was measured only once); specifically, the latent global relationship adjustment factor was regressed on the actor’s and partner’s *CD38* rs3796863 genotype.

Of note, both the predictor variables (i.e., actor and partner’s *CD38* rs3796863 status) and the dependent variables were characterized by missingness (which is not uncommon in studies that assess dyads overtime). To maximize the sample of participants for whom we had information about the rs3796863 status, we used full maximum likelihood estimator (FIML) with robust standard errors (MLR). FIML has been shown to produce unbiased estimates when missingness is assumed to have occurred at random^47,48^. As noted in the “Participants” section, participants’ data were included in the analyses as long as we had information from both partners on the dependent variable (i.e., ECR measures and global adjustment), and we allowed participants to have missing genetic information.

Gender differences were examined by comparing the fit of a model in which an estimate was permitted to differ between genders with the fit of a model in which the estimate was restricted to be equal in both genders. Model comparison was conducted using the rescaled − 2 log likelihood difference test, which is distributed as chi-squared with degrees of freedom equal to the rescaled difference in the number of parameters between models^67^. We used a liberal α-value of 0.10 to determine whether an estimate differed between genders. Pooled estimates are subsequently reported when no gender difference was found.

Lastly, we examined whether the within-partner and cross-partner associations were equal, that is, the presence of a “coupe pattern” (i.e., equal actor and partner effect). Kenny and Ledermann^68^ propose using a phantom variable *k*, which is a latent variable with no substantive meaning and no variance. *k* is defined as the ratio of the partner effect to the actor effect; a couple pattern is indicated when *k* takes a value of 1. Note that this is equivalent to testing whether the actor and partner effects are not statistically different. The − 2 log likelihood difference test can be used to examine whether fixing the actor and partner effects to be the same (i.e., *k* = 1) worsens model fit. A non-significant chi-square test value at α = 0.10 indicates the presence of a couple pattern; that is, the dependent variable is equally related to both the actor’s and partner’s *CD38* rs3796863 genotype.

### Power analysis

To address concerns about statistical power, we conducted two types of post-hoc power analyses for each of the final models reported in the Results section. Specifically, we used Monte Carlo simulations, one of the most widely used methods for computing statistical power in multilevel contexts (see e.g.,^49,69,70^). Specifically, we tested the following assumptions: if the model-estimated parameter values represent true population values, re-estimating the model using a set of randomly drawn samples of the same size from this population should yield significant values for the parameters of interest in a large proportion of these samples. Estimates from the final models were entered as starting values. For the ECR measures, we re-estimated the models using a set of 10,000 randomly drawn samples of 92 couples (184 participants) who provided, on average, ECR measures for 82 daily interactions (7,544 overall). For the relationship adjustment measure, we re-estimated the model using a set of 10,000 randomly drawn samples of 111 couples (222 participants). Summarized across 10,000 randomly drawn samples, the post hoc power estimates indicate the proportion of samples in which the estimates of interest would be statistically significant.

The post-hoc power estimate of the association between *CD38* rs3796863 and our primary outcome variable, communal behaviour, was 0.98; relative to a benchmark of 0.80, which is considered adequate power, this estimate suggests that our study was well-powered to test the association between *CD38* rs3796863 and communal behaviour in romantic relationships. This value suggests that we would find significant associations in about 98% of randomly drawn samples of the same size (assuming the effect is significant in the population). Similarly, our post-hoc power analysis suggests that our study was well-powered to examine the association between *CD38* rs3796863 and relationship adjustment (0.98), negative affect (0.97), and felt insecurity (0.81). Compared to the benchmark of 0.80, power was lower for positive affect (0.70), and for perceptions of partner’s communal behavior (0.66), indicating that our study had lower power to detect these associations assuming they are true in the population.

In addition to the Monte Carlo simulations, we used a tool specifically designed to estimate power in actor-partner interdependence models^50^. This tool uses formulas (see^46^) as an alternative to Monte Carlo simulations to compute statistical power. Results showed that one would need a sample of 57 dyads to have adequate power (i.e., 0.80) to detect the association between *CD38* rs3796863 and communal behavior that we found in our study (standardized estimate = 0.25). Similarly, sample sizes of 57, 62, and 101 dyads would be needed to detect the associations between *CD38* rs3796863 and relationship adjustment (0.25), negative affect (0.24), and felt insecurity (0.19), respectively, reported in our study. Our sample consisted of 92–111 dyads (sample size varied depending on the dependent variable); therefore, our sample size was sufficient to detect the effect size we report, with 80% power. Critically, even if we reduced our sample to those for whom we had genetic information from at least 1 partner (65 couples; 39 couples with complete data and 26 couples in which 1 partner had complete data), these numbers indicate that our sample size was sufficient to detect the effects we report.

## Supplementary information

Supplementary Information.

## Data Availability

These data cannot be deposited in a repository because participants did not consent to have their data made publicly available. We will, however, make the data available upon request for such purposes as confirming study results, conducting meta-analyses, etc. A Data Access Committee, consisting of Drs. Sadikaj, Moskowitz, Zuroff, Bartz, and Koestner, will assess requests for access to the data. Inquiries should be sent to Dr. Bartz at: jennifer.bartz@mcgill.ca.
