# Supplementary material for: CD38 is associated with communal behavior, partner perceptions, affect and relationship adjustment in romantic relationships
Source: Sci Rep. 2020 Aug 20;10:12926. doi: 10.1038/s41598-020-69520-y (PMC7441400; doi:10.1038/s41598-020-69520-y)
Supplement: Supplementary file 1 — Supplementary Information. [file 41598_2020_69520_MOESM1_ESM.docx]

Supplementary Information for

*CD38* is Associated with Communal Behavior, Partner Perceptions, Affect and Relationship Adjustment in Romantic Relationships

Gentiana Sadikaj, D. S. Moskowitz, David C. Zuroff and Jennifer A. Bartz^*^

Jennifer A. Bartz

Email: jennifer.bartz@mcgill.ca

**This PDF file includes:**

Supplemental Methods

*Genotyping Procedures*

Supplemental Tables

**Table S1:** *Associations Between the Actor’s and Partner’s CD38* rs3796863

*Genotype and Communal Behavior, Affect, Perception of the Partner’s Agentic Behavior, and Feelings of Security with the Romantic Partner*

**Table S2:** *Associations Between the Actor’s and Partner’s CD38 rs3796863*

*Genotype and Relationship Adjustment*

**Table S3:** *Sample Statistics and Correlations Among the Study Variables*

**Table S4:** *Genetics Sample Characteristics*

**Table S5:** *Results of Measurement Invariance Testing of the Hierarchical*

*Confirmatory Factory Analysis (CFA) Model of Global Relationship Adjustment*

**Table S6:** *Results of Measurement Invariance Testing of the CFA Model for*

*Dyadic Adjustment Scale*

**Table S7:** *Results of Measurement Invariance Testing of the CFA Model for*

*Autonomy Support Scale*

**Table S8:** *Results of Measurement Invariance Testing of the CFA Model for*

*Basic Need Satisfaction Scale*

**Table S9:** *Supplementary ECR Analyses in Sample Restricted to 65 Couples for*

*Whom at Least One Partner Had Genetic Information*

**Table S10:** *Supplementary Relationship Adjustment Analyses in Sample*

*Restricted to 74 Couples for Whom at Least One Partner Had Genetic Information*

Additional Information

*Statistical Power in Multi-Level Models: How Sample Size in each Level of a Multilevel Model Influences Statistical Power in General and in the Present Research*

**Supplemental Methods**

*Genotyping Procedures*

Genotyping was performed by Genome Quebec and was conducted in multiplex on 20ng of template genomic DNA in a 5uL reaction mixture containing: 0.1uL (0.5 U) HotStar Taq enzyme (QIAGEN), 0.625uL of 10X HotStar Buffer, 0.325uL of 25mM (total) MgCl2, 0.25uL of 10mM dNTP mix, 0.55uL of forward and reverse primer pool (1uM) and 1.15uL of water. The amplification cycling: 95c 15min, 45x (95c 20sec, 56c 30sec, 72c 60sec), 72c 3min, hold 4c. A few PCR reactions were conducted on QIAxcel (QIAGEN) to assess the amplification (1uL of PCR in 9uL of DNA Dilution Buffer (QIAGEN)). This was followed by a shrimp-alkaline-phosphatase treatment to remove the unused nucleotides (0.2uL of SAP Buffer, 0.3uL of SAP and 1.5uL of water). SAP cycling: 37c 40min, 85c 10min, hold 4c. Next, a primer extension reaction (iPLEX Gold) was performed with 0.94uL of extension primer mix, 0.2uL of iPlex Terminator, 0.2uL of iPlex Buffer, 0.041uL of iPlex Thermo Sequenase and 0.619uL of water. The products were desalted using 6mg of resin (Agena Bioscience) and spotted on a 384-point SpectroCHIP (Agena Bioscience) using a nanodispenser. The distinct masses were determined by MALDI-TOF mass-spectrometry and data was analyzed using MassARRAY Typer Analyser software. As noted, genotyping was unsuccessful for four participants: Genotyping failed for two of the saliva samples because the samples did not pass the call rate threshold of 80%; two other samples were deemed outliers compared to the cluster created by the other samples, and therefore no genotype could be attributed to these two samples.

Supplemental Tables

Table S1. *Associations Between the Actor’s and Partner’s CD38* rs3796863 *Genotype and Aggregated Communal Behavior, Affect, Perception of the Partner’s Agentic Behavior, and Feelings of Security with the Romantic Partner*

|  | **Gender**  **∆** | **Pooled across genders** | | | | **Women** | | | | **Men** | | | |
| --- | --- | --- | --- | --- | --- | --- | --- | --- | --- | --- | --- | --- | --- |
|  | *χ²* | *Unstnd*  *Estimate*  *(SE)* | *z* | *p* | *95% CI* | *Unstnd*  *Estimate*  *(SE)* | *z* | *p* | *95% CI* | *Unstnd*  *Estimate*  *(SE)* | *z* | *p* | *95% CI* |
| **Actor’s Gen. 🡪 Actor’s** |  |  |  |  |  |  |  |  |  |  |  |  |  |
| Com. Beh. | .67 | **-.069 (.024)** | **-2.859** | **.004** | **-.116,-.022** | -.052 (.034) | -1.512 | .131 | -.119, .015 | **-.098 (.038)** | **-2.539** | **.011** | **-.173,-.022** |
| Age. Beh. | **4.62*** |  |  |  |  | .033 (.027) | 1.227 | .220 | -.020, .086 | -.041 (.024) | -1.743 | .081 | -.088, .005 |
| Perc. Com. | .69 | **-.386 (.164)** | **-2.360** | **.018** | **-.707,-.065** | -.220 (.272) | -.810 | .418 | -.754, .313 | **-.569 (.261)** | **-2.178** | **.029** | **-1.082,-.057** |
| Perc. Age. | 1.30 | .243 (.258) | 0.942 | .346 | -.263, .748 | .033 (.310) | .108 | .914 | -.574, .641 | .591 (.358) | 1.650 | .099 | -.111, 1.292 |
| Neg. Affect | 2.63 | **.250 (.090)** | **2.761** | **.006** | **.072, .398** | .055 (.134) | .408 | .683 | -.208, .318 | **.351 (.121)** | **2.888** | **.004** | .113, .589 |
| Pos. Affect | 1.27 | **-.325 (.158)** | **-2.058** | **.040** | **-.635,-.015** | -.102 (.270) | -.376 | .707 | -.631, .428 | **-.508 (.206)** | **-2.463** | **.014** | **-.911,-.104** |
| Felt Insec. | 1.26 | **.220 (.099)** | **2.208** | **.027** | **.025, .414** | .139 (.115) | 1.205 | .228 | -.087, .365 | **.358 (.165)** | **2.173** | **.030** | **.035, .681** |
| Felt Sec. | 1.33 | -.187 (.177) | -1.062 | .288 | -.533, .159 | .024 (.278) | .086 | .932 | -.522, .569 | -.467 (.279) | -1.678 | .093 | -1.014, .079 |
| **Actor’s Gen. 🡪 Partner’s** |  |  |  |  |  |  |  |  |  |  |  |  |  |
| Com. Beh. | 2.05 | **-.055 (.023)** | **-2.404** | **.016** | **-.099, -.010** | -.011 (.036) | -.297 | .767 | -.082, .060 | **-.091 (.032)** | **-2.859** | **.004** | **-.153,-.029** |
| Age. Beh. | **9.38**** |  |  |  |  | .019 (.027) | .697 | .486 | -.034, .072 | **-.069 (.023)** | **-2.940** | **.003** | **-.115,-.023** |
| Perc. Com. | .19 | -.249 (.174) | -1.433 | .152 | -.590, .092 | -.368 (.329) | -1.121 | .262 | -1.013, .276 | -.164 (.258) | -.637 | .524 | -.670, .341 |
| Perc. Age. | .67 | .139 (.205) | 0.680 | .497 | -.262, .541 | -.069 (.304) | -.227 | .820 | -.664, .526 | .249 (.259) | .964 | .335 | -.258, .756 |
| Neg. Affect | 1.17 | **.190 (.084)** | **2.626** | **.024** | **.025, .329** | .001 (.163) | .006 | .995 | -.319, .321 | **.234 (.099)** | **2.357** | **.018** | **.039, .429** |
| Pos. Affect | **2.92^†^** |  |  |  |  | *-.344 (.312)* | *-1.101* | *.271* | *-.955, .268* | *.179 (.179)* | *.999* | *.318* | *-.172, .529* |
|  |  |  |  |  |  | -.400 (.314) | -1.274 | .203 | -1.015, .215 | .238 (.192) | 1.236 | .216 | -.139, .615 |
| Felt Insec. | .32 | .141 (.108) | 1.310 | .190 | -.070, .353 | .225 (.154) | 1.462 | .144 | -.077, .527 | .111 (.145) | .765 | .444 | -.173, .395 |
| Felt Sec. | 2.38 | -.060 (.173) | -0.349 | .727 | -.400, .279 | -.474 (.317) | -1.495 | .135 | -1.094, .147 | .190 (.243) | .780 | .435 | -.287, .666 |

*Note*. *N =* 92 couples (184 participants), between 7450 –7579 daily events. “Com. Beh.” = Communal Behavior; “Age. Beh.” = Agentic Behavior; “Perc. Com.” = Perceived Communal Behavior; “Perc. Age.” = Perceived Agentic Behavior; “Neg. Affect” = Negative Affect; “Pos. Affect” = Positive Affect; “Felt Insec.” = Felt Insecurity; “Felt Sec.” = Felt Security. The χ^2^ value comparing nested models is presented in the first column; a non-significant χ^2^ indicates the absence of a gender difference. Significant effects are in bold. **†***p* < .10; ** p* < .05; *** p* < .01; **** p* < .001.

The estimates presented in the “Pooled across genders” column are those pooled across men and women (i.e., when no gender difference was found). The estimates presented in the “Women” and “Men” columns are those obtained when the unrestricted APIM models were first estimated (i.e., when estimates were free to differ across gender); the estimates in italics in the same “Women” and “Men” columns are those found to be statistically different between genders.

Table S2. *Associations Between the Actor’s and Partner’s CD38 rs3796863 Genotype and Relationship Adjustment*

|  | **Gender**  **∆** | **Pooled across genders** | | | | **Women** | | | | **Men** | | | |
| --- | --- | --- | --- | --- | --- | --- | --- | --- | --- | --- | --- | --- | --- |
|  | *χ²* | *Unstnd*  *Estimate*  *(SE)* | *z* | *p* | *95% CIª* | *Unstnd*  *Estimate*  *(SE)* | *z* | *p* | *95% CI* | *Unstnd*  *Estimate*  *(SE)* | *z* | *p* | *95% CI* |
| **Actor’s Gen. 🡪 Actor’s** |  |  |  |  |  |  |  |  |  |  |  |  |  |
| Rel. Adjust. | 1.24 | **-.633 (.207)** | **-3.058** | **.002** | **-1.039, -.227** | -.445 (.298) | -1.492 | .136 | -1.029, .140 | **-.876 (.262)** | **-3.349** | **.001** | **-1.389, -.363** |
| **Actor’s Gen. 🡪 Partner’s** |  |  |  |  |  |  |  |  |  |  |  |  |  |
| Rel. Adjust. | 1.02 | **-.426 (.226)** | **-1.886** | **.059** | **-.869, .017** | **-.652 (.326)** | **-2.001** | **.045** | **-1.291, -.013** | -.262 (.283) | -.928 | .354 | -.816, .292 |

*Note*. *N =* 111 couples (222 participants). “Rel. Adjust.” = Relationship Adjustment. Model fit: (1) estimates free to vary across genders: χ² (155, N = 111) = 237.53, p = .001; CFI = .908; RMSEA = .069; SRMR = .121; (2) gender equality established: χ² (157, N = 111) = 238.61, p = .001; CFI = .909; RMSEA = .068; SRMR = .118.

The χ2 value comparing nested models is presented in the first column. Significant effects are in bold. †p < .10; * p < .05; ** p < .01; *** p < .001.

The estimates presented in the “Pooled across genders” column are those pooled across men and women (i.e., when no gender difference was found). The estimates presented in the “Women” and “Men” columns are those obtained when the unrestricted APIM models were first estimated (i.e., when estimates were free to differ across gender). “Unstnd” = Unstandardized; “CI” = Confidence interval.

**Table S3.** *Sample Statistics and Correlations Among the Study Variables*

| Variable | 1 | 2 | 3 | 4 | 5 | 6 | 7 | 8 | 9 | 10 | 11 | 12 | 13 | 14 | 15 |
| --- | --- | --- | --- | --- | --- | --- | --- | --- | --- | --- | --- | --- | --- | --- | --- |
| 1. M-Com Beh |  |  |  |  |  |  |  |  |  |  |  |  |  |  |  |
| 2. W-Com Beh | .31** |  |  |  |  |  |  |  |  |  |  |  |  |  |  |
| 3. M-Age Beh | .22** | .20* |  |  |  |  |  |  |  |  |  |  |  |  |  |
| 4. W-Age Beh | .14* | .18* | .01 |  |  |  |  |  |  |  |  |  |  |  |  |
| 5. M-Perc Com | .39*** | .36*** | .17 | .03 |  |  |  |  |  |  |  |  |  |  |  |
| 6.W-Perc Com | .32*** | .40*** | .03 | .12 | .65*** |  |  |  |  |  |  |  |  |  |  |
| 7. M-Perc Age | .15† | .17* | -.02 | .01 | .15 | .06 |  |  |  |  |  |  |  |  |  |
| 8.W-Perc Age | .12* | .12 | .01 | -.01 | .06 | .12 | .22* |  |  |  |  |  |  |  |  |
| 9. M-Neg Aff | -.26** | -.17*† | -.29** | .02 | -.45*** | -.31*** | .01 | -.09 |  |  |  |  |  |  |  |
| 10. W-Neg Aff | -.14† | -.26** | .02 | -.21** | -.28*** | -.38*** | -.1 | .01 | .45*** |  |  |  |  |  |  |
| 11. M-Pos Aff | .33*** | .34*** | .02 | .06 | .50*** | .35*** | .14 | .06 | -.14 | -.16* |  |  |  |  |  |
| 12. W-Pos Aff | .25*** | .50*** | .06 | .02 | .30*** | .52*** | .06 | .10 | -.14† | -.10† | .38*** |  |  |  |  |
| 13. M-Insec | -.19** | -.29*** | -.40*** | -.11 | -.52*** | -.40*** | .02 | -.05 | .79*** | .42*** | -.21** | -.20*** |  |  |  |
| 14. W-Insec | -.28*** | -.21** | -.15 | -.33*** | -.43*** | -.52*** | -.06 | .02 | .48*** | .74*** | -.26*** | -.17** | .63*** |  |  |
| 15. M-Sec | .40*** | .37*** | .18* | .12† | .56*** | .50*** | .12 | .08 | -.26** | -.22*** | .76*** | .38*** | -.46*** | -.41*** |  |
| 16. W-Sec | .34*** | .43*** | .16† | .14* | .51*** | .65*** | .10 | .10 | -.24** | -.23** | 0.47*** | .62*** | -.40*** | -.47*** | .66*** |
| 17. M-Aut | .143 | .22** | .38*** | .07 | .40*** | .27*** | -.12 | -.07 | -.45*** | -.20* | .17* | .19** | -.53*** | -.34*** | .30*** |
| 18. W-Aut | .19* | .14† | .09 | **.29***** | .27*** | .37*** | -.09 | -.09 | -.23* | -.38*** | .14† | .14* | -.31*** | -.61*** | .29*** |
| 19. M-Rel | .30** | .22* | .04 | .12† | .57*** | .45*** | .06 | -.04 | -.35*** | -.26** | .45*** | .27*** | -.49*** | -.42*** | .63*** |
| 20. W-Rel | .20* | .31*** | .15† | .12 | .45*** | .54*** | -.06 | .05 | -.29** | -.31*** | .32*** | .37*** | -.39*** | -.49*** | .49*** |
| 21. M-Comp | .17† | .12 | .29** | .02 | .41*** | .23** | -.03 | -.08 | -.38*** | -.08 | .29** | .14† | -.47*** | -.23** | .45*** |
| 22. W-Comp | .10 | .16† | .02 | .20* | .21* | .35*** | -.09 | -.02 | -.07 | -.29*** | .16† | .34*** | -.19** | -.44*** | .29*** |
| 23. M-Sup | .33*** | .34*** | .16 | .14† | .57*** | .36*** | .00 | -.05 | -.33*** | -.28*** | .33*** | .26** | -.47*** | -.39*** | .52*** |
| 24. W-Sup | .31*** | .34*** | .17† | .12 | .36*** | .52*** | -.06 | .00 | -.29*** | -.28*** | .33*** | .36*** | -.36*** | -.47*** | .46*** |
| 25. M-Sat | .23* | .27** | .04 | .12 | .47*** | .43*** | .02 | .01 | -.35** | -.24* | .44*** | .26*** | -.51*** | -.44*** | .59*** |
| 26. W-Sat | .02 | .23* | .14† | .03 | .43*** | .43*** | .02 | .02 | -.26* | -.28** | .30*** | .33*** | -.40*** | -.51*** | .47*** |
| 27. M-Con | .18* | .22* | .00 | .04 | .48*** | .36*** | -.01 | -.11 | -.28* | -.16 | .34*** | .30*** | -.34*** | -.29** | .40*** |
| 28. W-Con | .24** | .23* | .06 | .00 | .44*** | .44*** | .03 | -.01 | -.31* | -.24* | .42*** | .32*** | -.33** | -.42*** | .43*** |
| 29. M-Coh | .17† | .22* | .05 | .03 | .36*** | .22* | .20 | .07 | -.06 | .00 | .43*** | .41*** | -.15* | -.05 | .38*** |
| 30. W-Coh | .19* | .17† | .03 | .04 | .21* | .31*** | .08 | -.01 | .00 | .00 | .17 | .29*** | -.03 | -.14† | .29*** |
| 31. M-Affe | .22** | .20* | .01 | .07 | .42*** | .33*** | .00 | .03 | -.26** | -.32*** | .33*** | .22** | -.30*** | -.23** | .38*** |
| 32. W-Affe | .20* | .25*** | .10 | .01 | .37*** | .38*** | .04 | .00 | -.20† | -.39*** | .29*** | .29*** | -.24*** | -.33*** | .31*** |
| Mean | .33 | .27 | .12 | .16 | 8.63 | 8.60 | 7.16 | 7.16 | .55 | .63 | 3.35 | 3.27 | .57 | .57 | 4.54 |
| SD | .12 | .12 | .08 | .09 | .96 | 1.03 | .98 | 1.18 | .43 | .47 | .81 | 1.06 | .49 | .46 | .94 |

| Variable | 16 | 17 | 18 | 19 | 20 | 21 | 22 | 23 | 24 | 25 | 26 | 27 | 28 | 29 | 30 | 31 | 32 |
| --- | --- | --- | --- | --- | --- | --- | --- | --- | --- | --- | --- | --- | --- | --- | --- | --- | --- |
| 15. M-Sec |  |  |  |  |  |  |  |  |  |  |  |  |  |  |  |  |  |
| 16. W-Sec |  |  |  |  |  |  |  |  |  |  |  |  |  |  |  |  |  |
| 17. M-Aut | .29*** |  |  |  |  |  |  |  |  |  |  |  |  |  |  |  |  |
| 18. W-Aut | .47*** | .39*** |  |  |  |  |  |  |  |  |  |  |  |  |  |  |  |
| 19. M-Rel | .50*** | .58*** | .44*** |  |  |  |  |  |  |  |  |  |  |  |  |  |  |
| 20. W-Rel | .61*** | .45*** | .54*** | .64*** |  |  |  |  |  |  |  |  |  |  |  |  |  |
| 21. M-Comp | .32*** | .65*** | .23*** | .67*** | .38*** |  |  |  |  |  |  |  |  |  |  |  |  |
| 22. W-Comp | .41*** | .21*** | .56*** | .35*** | .58*** | .18* |  |  |  |  |  |  |  |  |  |  |  |
| 23. M-Sup | .47*** | .60*** | .46*** | .68*** | .58*** | .60*** | .35*** |  |  |  |  |  |  |  |  |  |  |
| 24. W-Sup | .62*** | .46*** | .55*** | .43*** | .64*** | .38*** | .52*** | .46*** |  |  |  |  |  |  |  |  |  |
| 25. M-Sat | .49*** | .55*** | .45*** | .82*** | .64*** | .61*** | .39*** | .63*** | .49*** |  |  |  |  |  |  |  |  |
| 26. W-Sat | .56*** | .45*** | .51*** | .62*** | .76*** | .45*** | .53*** | .49*** | .58*** | .74*** |  |  |  |  |  |  |  |
| 27. M-Con | .37*** | .46*** | .51*** | .54*** | .41*** | .42*** | .27*** | .54*** | .37*** | .56*** | .43*** |  |  |  |  |  |  |
| 28. W-Con | .47*** | .37*** | .51*** | .49*** | .62*** | .36*** | .43*** | .45*** | .61*** | .53*** | .62*** | .56*** |  |  |  |  |  |
| 29. M-Coh | .32*** | .30*** | .20* | .50*** | .33*** | .41*** | .24** | .42*** | .31** | .51*** | .42*** | .49*** | .43*** |  |  |  |  |
| 30. W-Coh | .34*** | .19** | .26*** | .31** | .44*** | .24** | .33*** | .29** | .37*** | .40*** | .44*** | .33*** | .52*** | .43*** |  |  |  |
| 31. M-Affe | .40*** | .33*** | .26** | .58*** | .42*** | .32** | .20* | .41*** | .36*** | .52*** | .46*** | .42*** | .33*** | .38*** | .14† |  |  |
| 32. W-Affe | .41*** | .29** | .34*** | .47*** | .61*** | .24** | .31*** | .40*** | .43*** | .51*** | .54*** | .30*** | .53*** | .31*** | .36**** | .59*** |  |
| Mean | 4.68 | 5.95 | 6.31 | 6.09 | 6.07 | 6.00 | 5.87 | 5.36 | 5.50 | 3.99 | 3.97 | 3.78 | 3.81 | 3.67 | 3.66 | 2.17 | 2.25 |
| SD | 1.08 | 1.02 | 1.02 | 1.04 | 1.11 | 1.01 | 1.15 | .98 | 1.10 | .55 | .64 | .54 | .47 | .60 | .61 | .54 | .45 |

*Note*. *N* = 92 couples (184 participants), 7579 reported social interactions.

“M” = Men’s variables; “W” = Women’s variables; “Com Beh” = Communal Behavior; “Age Beh” = Agentic Behavior; “Perc Com” = Perceived Communal Behavior; “Perc Age” = Perceived Agentic Behavior; “Neg Aff” = Negative Affect; “Pos Aff” = Positive Affect; “Insec” = Felt Insecurity; “Sec” = Felt Security; “Aut” = BNSR Autonomy; “Rel” = BNSR Relatedness; “Comp” = BNSR Competence; “Sup” = Autonomy Support; “Sat” = DAS Satisfaction; “Con” = DAS Consensus; “Coh” = DAS Cohesion; “Affe” = DAS Affection; “ICC” = Intra-class correlation

^1^ Means and standard deviations across all reported interactions.

**†***p*  < .10; **p*  < .05; *** p* < .01; **** p* < .001;

**Table S4.** *Genetics Sample Characteristics*

| Variable | | *Mean/*  *Proportion* | *Standard*  *Deviation* | *Range* |
| --- | --- | --- | --- | --- |
| Age (years) | | 33.95 | 7.09 | 23.00 – 59.00 |
| Relationship length (months)^a^ | | 58.19 | 69.58 | 8.00 – 384.00 |
| Cohabitation length (months)^1a^ | | 42.66 | 67.28 | 7.00 – 379.00 |
| Weekly working hours | | 39.28 | 8.20 | 25.00 – 80.00 |
| Women | | 55.00% |  |  |
| Education | |  |  |  |
|  | Less than high school | 0.85% |  |  |
|  | High school or trade school | 8.55% |  |  |
|  | 1 year of college/university or more | 29.91% |  |  |
|  | University graduate | 36.75% |  |  |
|  | Postgraduate degree | 23.08% |  |  |
|  | Doctoral degree | 0.85% |  |  |
| Ethnicity² | |  |  |  |
|  | Caucasian/White | 70.34% |  |  |
|  | Asian | 11.02% |  |  |
|  | Black | 6.78% |  |  |
|  | Hispanic | 9.32% |  |  |
|  | Native | 1.69% |  |  |
|  | Middle-Eastern | 3.39% |  |  |
|  | Other | 2.54% |  |  |
|  | North Africa | 1.69% |  |  |
|  | Did not specify | 0.85% |  |  |

*Note*. *N* = 118 participants

^a^ Information reported prior to the start of the ECR procedure; ¹ Information was missing for 1 participant. ² Percentages add to more than 100; 1 participant reported Caucasian/White and Hispanic ethnicities; 1 participant reported Caucasian/White, Hispanic, Black, and Native ethnicities; 1 participant reported Caucasian/White, Black, and Native ethnicities; and 1 participant reported Caucasian/White and North African ethnicities.

Table S5. *Results of Measurement Invariance Testing of the Hierarchical Confirmatory Factory Analysis (CFA) Model of Relationship Adjustment*

| Parameter | Gender ∆ |  | *Pooled*  *Across*  *Genders* | |  |  | *Women* | |  | *Men* | | |  |  |
| --- | --- | --- | --- | --- | --- | --- | --- | --- | --- | --- | --- | --- | --- | --- |
|  | *χ²* |  | *Unstnd*  *Estimate*  *(SE)* |  |  |  | *Unstnd*  *Estimate*  *(SE)* |  |  | *Unstnd*  *Estimate*  *(SE)* |  | |  |  |
| *Factor Loadings:* |  |  |  | |  |  |  |  |  |  |  |  |  |  |
| DAS | .95 |  | 4.96 (3.88) | |  |  | 4.79 (3.42) |  |  | 4.74 (3.38) |  |  |  |  |
| AS | 2.31 |  | **1.03 (0.13)***** | |  |  | **0.96 (0.13)***** |  |  | **1.14 (0.16)***** |  |  |  |  |
| BNSR | .04 |  | 4.46 (2.72) | |  |  | 4.62 (2.97) |  |  | 4.61 (2.97) |  |  |  |  |
| DAS = BNSR | .01 |  | **4.69 (1.54)**** | |  |  |  |  |  |  |  |  |  |  |

*Note*. *N =* 111 couples (222 participants). **†***p* < .10; ** p* < .05; *** p* < .01; **** p* < .001

The χ2 value comparing nested models is presented in the first column. Significant effects are in bold. †p < .10; * p < .05; ** p < .01; *** p < .001.

The estimates presented in the “Pooled across genders” column are those pooled across men and women (i.e., when no gender difference was found). The estimates presented in the “Women” and “Men” columns are those obtained when the unrestricted APIM models were first estimated (i.e., when estimates were free to differ across gender). “DAS = BNSR” = Testing whether the factor loadings for the latent DAS and BNSR factors were equal.

“Unstnd” = Unstandardized.

**Table S6.** *Results of Measurement Invariance Testing of the CFA Model for Dyadic Adjustment Scale*

| Model | LL | SC | P | Reference  model | Δχ² | Δ*df* | RMSEA (90% CI) | CFI | SRMR | ΔRMSEA | ΔCFI | ΔSRMR |
| --- | --- | --- | --- | --- | --- | --- | --- | --- | --- | --- | --- | --- |
| 1. Configural | -551.54 | 1.24 | 25 | 0 | - |  | .143 (.104,.183) | .876 | .057 | - | - | - |
| 2. Metric | -553.82 | 1.27 | 19 | 1 | 3.96 | 6 | .120 (.084,.156) | .885 | .100 | -.023 | .090 | .043 |
| 3. Scalar | -554.81 | 1.35 | 16 | 2 | 1.21 | 3 | .112 (.078,.147) | .886 | .103 | -.008 | .001 | .003 |
| 4. Residual Variances | -555.92 | 1.46 | 12 | 3 | 3.09 | 4 | .104 (.071,.137) | .889 | .129 | -.008 | .003 | .026 |

*Note*. *N =* 111 couples (222 participants). “Configural” = Base model, estimates are free to vary across gender; “Metric” = Model in which factor loadings are fixed to equality across gender; “Scalar” = Model in which intercepts (subscale means) are fixed to equality across gender; “Residual Variances” = Model in which subscale residual variance are fixed to equality across gender; “LL” = Loglikelihood; “SC” = Scaling Correcting Factor; “P” = Number of estimated parameters; “Reference model” = Order of nested models with higher numbers indicating more restricted models; “Δχ²” = Difference in chi-square value between nested models; “Δ*df*” = Difference in degrees of freedom between nested models; “RMSEA” = Root Mean Square Error of Approximation; “CFI” = Comparative Fit Index; “SRMR” = Standardized Root Mean Square Residual; “ΔRMSEA” = Difference in RMSEA between nested models; “ΔCFI” = Difference in CFI between nested models; “ΔSRMR” = Difference in SRMR between nested models. With the exception of the intercept of Affection Subscale, all other estimates were found to be invariant across gender. Equality across factor loadings of Affection, Cohesion, and Consensus Subscales was found.

Significant effects are in bold. †p < .10; * p < .05; ** p < .01; *** p < .001.

**Table S7.** *Results of Measurement Invariance Testing of the CFA Model for Autonomy Support Scale*

| Model | LL | SC | P | Reference  model | Δχ² | Δ*df* | RMSEA (90% CI) | CFI | SRMR | ΔRMSEA | ΔCFI | ΔSRMR |
| --- | --- | --- | --- | --- | --- | --- | --- | --- | --- | --- | --- | --- |
| 1. Configural | -316.25 | 1.05 | 5 | 0 | - |  | .000 (.000,.000) | 1.000 | .000 | - | - | - |
| 2. Metric | -317.33 | 1.12 | 4 | 1 | **2.78**† | 1 | .127 (.000,.314) | .867 | .222 | .127 | -.133 | .222 |
| 3. Scalar | -316.94 | 1.05 | 4 | 1 | 1.33 | 1 | .055 (.000,.266) | .975 | .041 | .055 | -.025 | .041 |
| 4. Residual Variances |  |  |  |  |  |  |  |  |  |  |  |  |

*Note*. *N =* 111 couples (222 participants). “Configural” = Base model, estimates are free to vary across gender; “Metric” = Model in which factor loadings are fixed to equality across gender; “Scalar” = Model in which intercepts (subscale means) are fixed to equality across gender; “Residula Variances” = Model in which subscale residual variance are fixed to equality across gender; “LL” = Loglikelihood; “SC” = Scaling Correcting Factor; “P” = Number of estimated parameters; “Reference model” = Order of nested models with higher numbers indicating more restricted models; “Δχ²” = Difference in chi-square value between nested models; “Δ*df*” = Difference in degrees of freedom between nested models; “RMSEA” = Root Mean Square Error of Approximation; “CFI” = Comparative Fit Index; “SRMR” = Standardized Root Mean Square Residual; “ΔRMSEA” = Difference in RMSEA between nested models; “ΔCFI” = Difference in CFI between nested models; “ΔSRMR” = Difference in SRMR between nested models. Factor loading was different across gender; the was found to be invariant across gender.

Significant effects are in bold. †p < .10; * p < .05; ** p < .01; *** p < .001.

**Table S8.** *Results of Measurement Invariance Testing of the CFA Model for Basic Need Satisfaction Scale*

| Model | LL | SC | P | Reference  model | Δχ² | Δ*df* | RMSEA (90% CI) | CFI | SRMR | ΔRMSEA | ΔCFI | ΔSRMR |
| --- | --- | --- | --- | --- | --- | --- | --- | --- | --- | --- | --- | --- |
| 1. Configural | -862.98 | 1.46 | 19 | 0 | - |  | .139 (.080,.202) | .899 | .072 | - | - | - |
| 2. Metric | -866.26 | 1.31 | 14 | 1 | 3.48 | 5 | .096 (.041,.149) | .922 | .132 | -.043 | .023 | .060 |
| 3. Scalar | -866.26 | 1.38 | 12 | 2 | .02 | 2 | .088 (.033,.138) | .924 | .132 | -.008 | .002 | .000 |
| 4. Residual Variances | -867.55 | 1.32 | 9 | 3 | 1.65 | 3 | .076 (.013,.123) | .933 | .138 | -.012 | .009 | .006 |

*Note*. *N =* 111 couples (222 participants). “Configural” = Base model, estimates are free to vary across gender; “Metric” = Model in which factor loadings are fixed to equality across gender; “Scalar” = Model in which intercepts (subscale means) are fixed to equality across gender; “Residula Variances” = Model in which subscale residual variance are fixed to equality across gender; “LL” = Loglikelihood; “SC” = Scaling Correcting Factor; “P” = Number of estimated parameters; “Reference model” = Order of nested models with higher numbers indicating more restricted models; “Δχ²” = Difference in chi-square value between nested models; “Δ*df*” = Difference in degrees of freedom between nested models; “RMSEA” = Root Mean Square Error of Approximation; “CFI” = Comparative Fit Index; “SRMR” = Standardized Root Mean Square Residual; “ΔRMSEA” = Difference in RMSEA between nested models; “ΔCFI” = Difference in CFI between nested models; “ΔSRMR” = Difference in SRMR between nested models. With the exception of the intercept of Autonomy Subscale, all other estimates were found to be invariant across gender. Equality across factor loadings of all Subscales was found.

Significant effects are in bold. †p < .10; * p < .05; ** p < .01; *** p < .001.

**Table S9.** *Associations Between the Actor’s and Partner’s CD38* rs3796863 *Genotype and Communal and Agentic Behavior, Perception of the Partner’s Communal and Agentic Behavior, Affect and Feelings of Security with the Romantic Partner in Sample Restricted to 65 Couples for Whom at Least One Partner Had Genetic Information*

|  | Gender  ∆ |  | Pooled across genders | |  |  |  | | Women | |  |  |  |  |  | | | | Men |
| --- | --- | --- | --- | --- | --- | --- | --- | --- | --- | --- | --- | --- | --- | --- | --- | --- | --- | --- | --- |
|  | *χ²* |  | *Unstnd*  *Estimate*  *(SE)* | *z* | *p* | *95% CIª* |  | | *Unstnd*  *Estimate*  *(SE)* | *z* | *p* | *95% CI* |  | *Unstnd*  *Estimate*  *(SE)* | *z* | *p* | | | *95% CI* |
| Actor’s Genotype 🡪 Actor’s |  |  |  |  |  |  |  | |  |  |  |  |  |  |  |  | | |  |
| Communal Behavior | .48 |  | **-.069 (.023)** | **-2.947** | **.003** | **-.116,-.022** |  | | -.054 (.032) | -1.672 | .095 | -.117, .009 |  | **-.091 (.038)** | **-2.400** | **.016** | | | **-.166,-.017** |
| Agentic Behavior | **3.77^†^** |  |  |  |  |  |  | | .032 (.029) | 1.222 | .262 | -.024, .088 |  | -.041 (.024) | -1.692 | .091 | | | -.088, .006 |
| Perception of Communal Behavior | .69 |  | **-.371 (.163)** | **-2.273** | **.023** | **-.691,-.051** |  | | -.207 (.265) | -.780 | .435 | -.726, .313 |  | **-.552 (.262)** | **-2.105** | **.035** | | | **-1.067,-.038** |
| Perception of Agentic Behavior | 1.13 |  | .301 (.290) | 1.039 | .299 | -.267, .868 |  | | .071 (.349) | .204 | .838 | -.613, .756 |  | .621 (.381) | 1.632 | .103 | | | -.125, 1.367 |
| Negative Affect | **3.58^†^** |  |  |  |  |  |  | | *-.008 (.132)* | *-.062* | *.950* | *-.266, .250* |  | ***.396 (.111)*** | ***3.575*** | ***.000*** | | | ***.179, .614*** |
|  |  |  |  |  |  |  |  | | .019 (.131) | .148 | .882 | -.238, .277 |  | **.342 (.116)** | **2.942** | **.003** | | | **.114, .569** |
| Positive Affect | 1.44 |  | **-.323 (.157)** | **-2.049** | **.040** | **-.631,-.014** |  | | -.074 (.274) | -.270 | .787 | -.611, .463 |  | **-.513 (.217)** | **-2.367** | **.018** | | | **-.938,-.088** |
| Felt Insecurity | 1.30 |  | **.211 (.097)** | **2.183** | **.029** | **.022, .400** |  | | .133 (.110) | 1.215 | .224 | -.082, .348 |  | **.355 (.175)** | **2.024** | **.043** | | | **.011, .699** |
| Felt Security | 1.42 |  | -.184 (.181) | -1.022 | .307 | -.538, .169 |  | | .039 (.299) | .130 | .897 | -.548, .626 |  | -.483 (.284) | -1.704 | .088 | | | -1.014, .073 |
|  |  |  |  |  |  |  |  | |  |  |  |  |  |  |  |  | | |  |
| Actor’s Genotype 🡪 Partner’s |  |  |  |  |  |  |  | |  |  |  |  |  |  |  |  | | |  |
| Communal Behavior | 1.75 |  | **-.056 (.022)** | **-2.605** | **.009** | **-.099, -.014** |  | | -.016 (.036) | -.441 | .659 | -.086, .054 |  | **-.087 (.031)** | **-2.835** | **.005** | | | **-.148,-.027** |
| Agentic Behavior | **6.88**** |  |  |  |  |  |  | | .018 (.029) | .624 | .533 | -.038, .074 |  | **-.068 (.024)** | **-2.830** | **.005** | | | **-.115,-.021** |
| Perception of Communal Behavior | .19 |  | -.227 (.172) | -1.325 | .185 | -.564, .109 |  | | -.333 (.329) | -1.013 | .311 | -.977, .311 |  | -.154 (.250) | -.618 | .537 | | | -.643, .335 |
| Perception of Agentic Behavior | .39 |  | .187 (.231) | 0.810 | .418 | -.265, .639 |  | | .001 (.342) | .004 | .997 | -.669, .672 |  | .265 (.278) | .954 | .340 | | | -.280, .811 |
| Negative Affect | 1.69 |  | .145 (.084) | 1.722 | .085 | -.020, .311 |  | | -.051 (.156) | -.329 | .742 | -.357, .254 |  | **.225 (.098)** | **2.292** | **.022** | | | **.033, .418** |
| Positive Affect | **3.10^*^** |  |  |  |  |  |  | | *-.345 (.309)* | *-1.118* | *.263* | *-.950, .260* |  | *.136 (.175)* | *.774* | *.439* | | | *-.208, .479* |
|  |  |  |  |  |  |  |  | | -.438 (.314) | -1.392 | .164 | -1.054, .179 |  | .224 (.198) | 1.320 | .258 | | | -.164, .613 |
| Felt Insecurity | .28 |  | .134 (.099) | 1.356 | .175 | -.060, .328 |  | | .214 (.151) | 1.416 | .157 | -.082, .511 |  | .108 (.139) | .775 | .438 | | | -.165, .381 |
| Felt Security | 2.39 |  | -.063 (.177) | -0.354 | .723 | -.410, .285 |  | | -.523 (.344) | -1.522 | .128 | -1.197, .151 |  | .175 (.245) | .714 | .475 | | | -.305, .655 |
|  |  |  |  |  |  |  |  | |  |  |  |  |  |  |  |  | | |  |
|  | Actor vs. Partner  ∆ |  | Pooled across actor and partner | | | |  | | Within-partner | | | |  | Cross-partner | | | | | |
| Couple Pattern Present? | *χ²* |  | *Unstnd*  *Estimate*  *(SE)* | *z* | *P* | *95% CIª* |  | | *Unstnd*  *Estimate*  *(SE)* | *z* | *P* | *95% CIª* |  | *Unstnd*  *Estimate*  *(SE)* | *z* | | *p* | | *95% CIª* |
| Communal Behavior | .29 |  | **-.062 (.019)** | **-3.212** | **.001** | **-.100,-.024** |  | |  |  |  |  |  |  |  | |  | |  |
| Agentic Behavior |  |  |  |  |  |  |  | |  |  |  |  |  |  |  | |  | |  |
| Women | .54 |  | .026 (.023) | 1.165 | .244 | -.018, .071 |  | |  |  |  |  |  |  |  | |  | |  |
| Men | .16 |  | **-.056 (.015)** | **-3.633** | **.000** | **-.087,-.026** |  | |  |  |  |  |  |  |  | |  | |  |
| Perception of Communal Behavior | .73 |  | **-.303 (.142)** | **-2.139** | **.032** | **-.581,-.025** |  | |  |  |  |  |  |  |  | |  | |  |
| Perception of Agentic Behavior | .18 |  | .226 (.218) | 1.034 | .301 | -.202, .653 |  | |  |  |  |  |  |  |  | |  | |  |
| Negative Affect |  |  |  |  |  |  |  | |  |  |  |  |  |  |  | |  | |  |
| Women | 1.45 |  | .108 (.083) | 1.293 | .196 | -.056, .271 |  | |  |  |  |  |  |  |  | |  | |  |
| Men | **3.91*** |  |  |  |  |  |  | | **.381 (.111)** | **3.422** | **.001** | **.163, .600** |  | .108 (.083) | 1.293 | | .196 | | -.056, .271 |
| Positive Affect |  |  |  |  |  |  |  | |  |  |  |  |  |  |  | |  | |  |
| Women | .01 |  | **-.326 (.159)** | **-2.058** | **.040** | **-.637,-.016** |  | |  |  |  |  |  |  |  | |  | |  |
| Men | **7.66**** |  |  |  |  |  |  | | **-.326 (.159)** | **-2.058** | **.040** | **-.637,-.016** |  | .133 (.173) | .770 | | .441 | | -.206, .472 |
| Felt Insecurity | 1.03 |  | **.173 (.087)** | **1.988** | **.047** | **.002, .344** | |  | |  |  |  |  |  |  |  | |  |  |
| Felt Security | .88 |  | -.113 (.167) | -0.675 | .500 | -.441, .215 | |  | |  |  |  |  |  |  |  | |  |  |

*Note*. *N =* 65 couples (130 participants), between 5231 daily events. The χ^2^ value comparing nested models is presented in the first column. Significant effects are in bold. **†***p* < .10; ** p* < .05; *** p* < .01; **** p* < .001. The estimates presented in the “Pooled across genders” column are those pooled across men and women (i.e., when no gender difference was found). The estimates presented in the “Women” and “Men” columns are those obtained when the unrestricted APIM models were first estimated (i.e., when estimates were free to differ across gender); the estimates in italics in the same “Women” and “Men” columns are those found to be statistically different between genders. The estimates presented in the “Pooled across actor and partner” column are those pooled across actor and partner associations (i.e., when a couple pattern was present).“Unstnd” = Unstandardized; “CI” = Confidence interval.

**Table S10.** *Associations Between the Actor’s and Partner’s CD38 rs3796863 Genotype and Relationship Adjustment in a Sample Restricted to 74 Couples for Whom at Least One Partner Had Genetic Information*

|  | Gender  ∆ | Pooled across genders | | | | | Women | | | | | | Men | | | | |  |
| --- | --- | --- | --- | --- | --- | --- | --- | --- | --- | --- | --- | --- | --- | --- | --- | --- | --- | --- |
|  | *χ²* | *Unstnd*  *Estimate*  *(SE)* | *z* | *p* | *95% CIª* | | *Unstnd*  *Estimate*  *(SE)* | | *z* | *p* | *95% CI* | | *Unstnd*  *Estimate*  *(SE)* | | *z* | *p* | *95% CI* |  |
| **Actor’s Gen. 🡪 Actor’s** |  |  |  |  |  | |  | |  |  |  | |  | |  |  |  |  |
| Rel. Adjust. | 1.12 | **-.625 (.205)** | **-3.056** | **.002** | **-1.026, -.224** | | -.452 (.290) | | -1.562 | .118 | -1.020, .115 | | **-.860 (.268)** | | **-3.209** | **.001** | **-1.386, -.335** |  |
| **Actor’s Gen. 🡪 Partner’s** |  |  |  |  |  | |  | |  |  |  | |  | |  |  |  |  |
| Rel. Adjust. | 1.35 | **-.410 (.224)** | **-1.829** | **.067** | **-.849, .029** | | **-.671 (.315)** | | **-2.130** | **.033** | **-1.288, -.053** | | -.227 (.285) | | -.796 | .426 | -.786, .332 |  |
| Couple Pattern Present? | Actor vs. Partner  ∆ | Pooled across actor and partner | | | | | Within-partner | | | | | | Cross-partner | | | | |  |
|  | *χ²* | *Unstnd*  *Estimate*  *(SE)* | *z* | *p* | *95% CIª* | | *Unstnd*  *Estimate*  *(SE)* | | *z* | *p* | *95% CI* | | *Unstnd*  *Estimate*  *(SE)* | | *z* | *p* | *95% CI* |  |
| Rel. Adjust. | 1.29 | **-.539 (.199)** | **-2.708** | **.007** | **-.929, -.149** |  | |  |  |  |  |  | |  |  |  |  |  |

*Note*. *N =* 74 couples (148 participants). “Rel. Adjust.” = Relationship Adjustment. Model fit: (1) estimates free to vary across genders: χ² (155, N = 74) = 224.40, p = .001; CFI = .902; RMSEA = .078; SRMR = .148; (2) gender equality established: χ² (157, N = 74) = 225.61, p = .001; CFI = .903; RMSEA = .077; SRMR = .148; and (3) couple pattern established: χ² (158, N = 74) = 227.48, p = .001; CFI = .902; RMSEA = .077; SRMR = .149..

The χ2 value comparing nested models is presented in the first column. Significant effects are in bold. †p < .10; * p < .05; ** p < .01; *** p < .001.

The estimates presented in the “Pooled across genders” column are those pooled across men and women (i.e., when no gender difference was found). The estimates presented in the “Women” and “Men” columns are those obtained when the unrestricted APIM models were first estimated (i.e., when estimates were free to differ across gender). The estimates presented in the “Pooled across actor and partner” column are those pooled across actor and partner associations (i.e., when a couple pattern was present). “Unstnd” = Unstandardized; “CI” = Confidence interval.

**Additional Information**

*Statistical Power in Multi-Level Models: How Sample Size in each Level of a Multilevel Model Influences Statistical Power in General and in the Present Research*

As noted in the Main Text, one of the strengths of this research is our use of intensive repeated measurement methods, which mitigates some of the limitations of our sample size. It is well-established that aggregating measurements across occasions and situations, as it is done in our study, increases measurement reliability; in this way, our study, as are other studies using repeated measurements designs, is characterized by enhanced statistical power (see, for example, Mathieu, Aguinis, Culpepper, & Chen, 2012). As Arend and Schäfer (2019) demonstrate, as the number of repeated measurements (e.g., event-level, L1) increase, one is able to detect, with the same degree of power, smaller sized effects at both the event-level (L1) and person-level (L2). In other words, as the L1 sample increases, statistical power also increases (see Tables 5, 6 and 7 of their paper; also see Discussion, p. 17).

Here, it is worth noting that in contrast to single-level analyses, calculating power in multilevel models is a complex issue due to the number of parameters involved in determining power—specifically, different sample sizes at each level, and different variances and covariances between variables at each level. Arend and Schäfer’s (2019) paper offers a guide to researchers to approximate power estimates in multi-level model studies. Using their simulation data, researchers can estimate the minimum effect size their study could detect, with power = .80, given the sample size at each level, and intra-class correlations (*ICC*). In our study, participants completed, on average, ECR forms on 55.44 interactions (*SD* = 18.89, *Range* = 10 – 127) daily interactions (L1 measurements) with their partner. Consulting Table 6 in Arend and Shäfer’s paper, and inputting the relevant parameter, i.e., *ICC* from our study, one can see that the minimum detectible L2 effect size decreases when one goes from an L1 sample of 3 to an L1 sample of 30 (the maximum L1 sample size presented in Arend and Schäfer’s Table 6).

Take, for example, communal behavior (*ICC* = .10)—one of the two primary outcomes in our study (the other being Relationship Adjustment). Given our L2 sample of 92, with an L1 sample of 3, one is only able to detect a relatively large effect size of .58, but when one increases the L1 sample to 30, one is able to detect a small to medium effect size of .34 with 80% power. Note that we actually had an L1 sample almost twice that size (i.e., 55); although Arend and Schäfer’s Table 6 does not provide estimates for L1 samples of 55, one can assume that power would continue to increase. The *ICCs* for most of the other ECR measures are of medium size; the information in the same Table suggests that with an L2 sample size of 90 and an L1 sample size of 30 one is able to detect a small to medium effect size of .30 with 80% power. Our effect sizes ranged from small to medium (.19 to .25); these analyses thus suggest that we were adequately powered to detect the effects we report.

References:

Arend, M. G., & Schäfer, T. (2019). Statistical power in two-level models: A tutorial based on Monte Carlo simulation.Psychological Methods, 24(1), 1–19. [https://doi.org/10.1037/met0000195](https://psycnet.apa.org/doi/10.1037/met0000195)

Mathieu, J. E., Aguinis, H., Culpepper, S. A., & Chen, G. (2012). Understanding and estimating the power to detect cross-level interaction effects in multilevel modeling. Journal of Applied Psychology, 97(5), 951–966. [https://doi.org/10.1037/a0028380](https://psycnet.apa.org/doi/10.1037/a0028380)
